# Supplementary material for: Comprehensive Genome-Wide Analysis and Expression Pattern Profiling of PLATZ Gene Family Members in Solanum Lycopersicum L. under Multiple Abiotic Stresses
Source: Plants (Basel). 2022 Nov 15;11(22):3112. doi: 10.3390/plants11223112 (PMC9697139; doi:10.3390/plants11223112)
Supplement: Supplementary file 1 [file plants-11-03112-s001.zip › Figure S7. Homology in the cDNA sequences of SlPLATZ genes..pptx]

## Slide 1
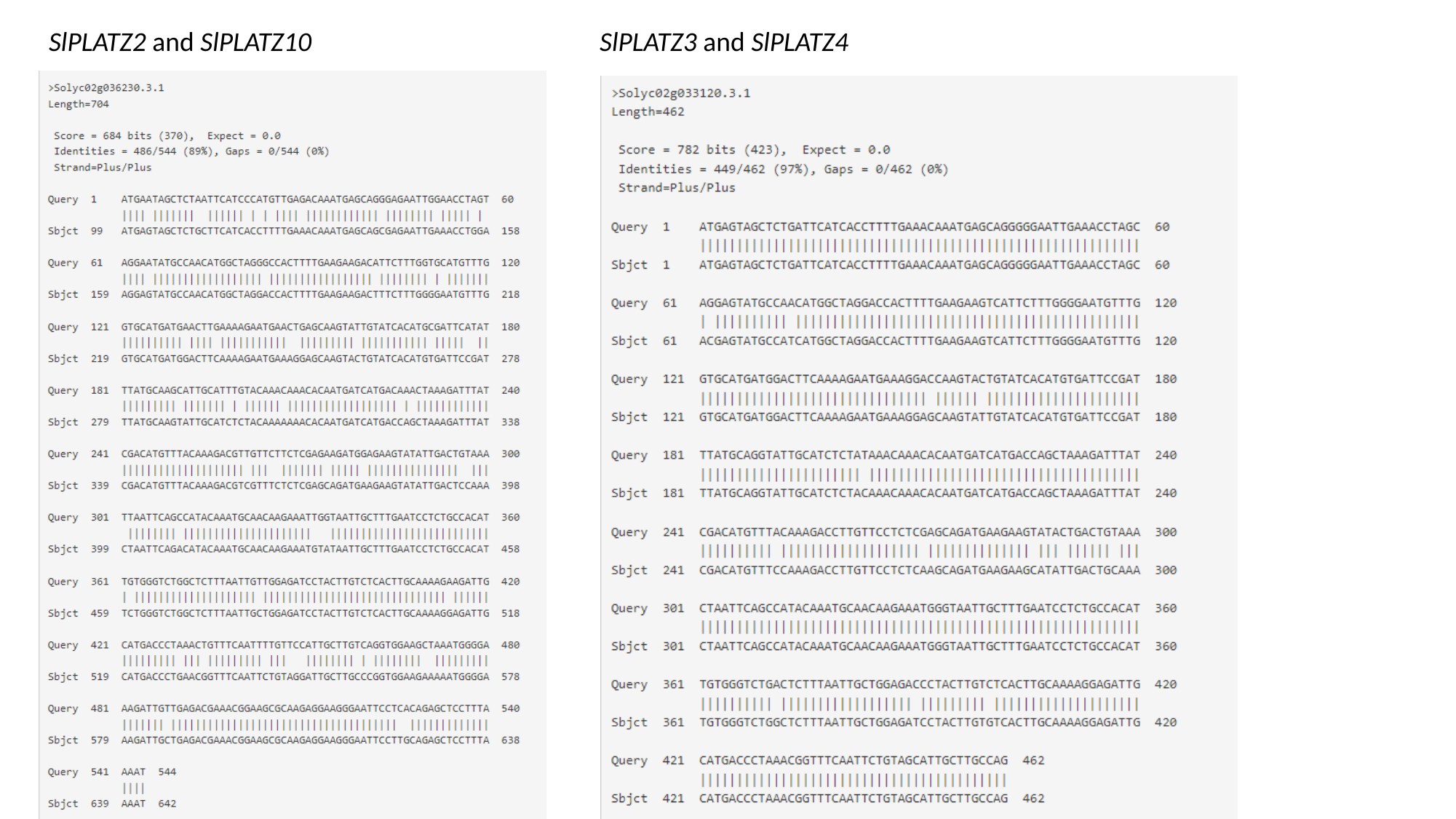

SlPLATZ2 and SlPLATZ10
SlPLATZ3 and SlPLATZ4

## Slide 2
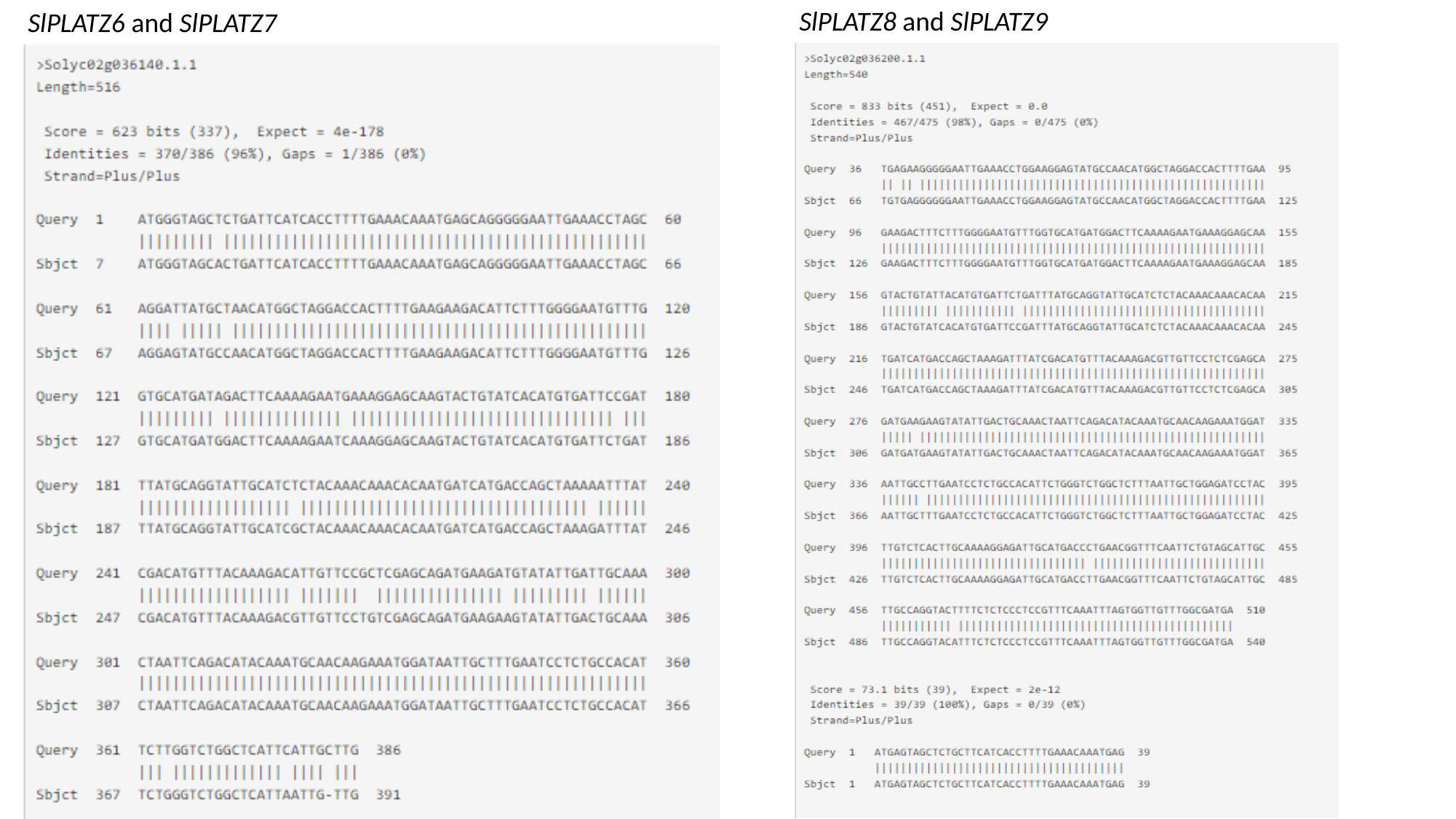

SlPLATZ6 and SlPLATZ7
SlPLATZ8 and SlPLATZ9

## Slide 3
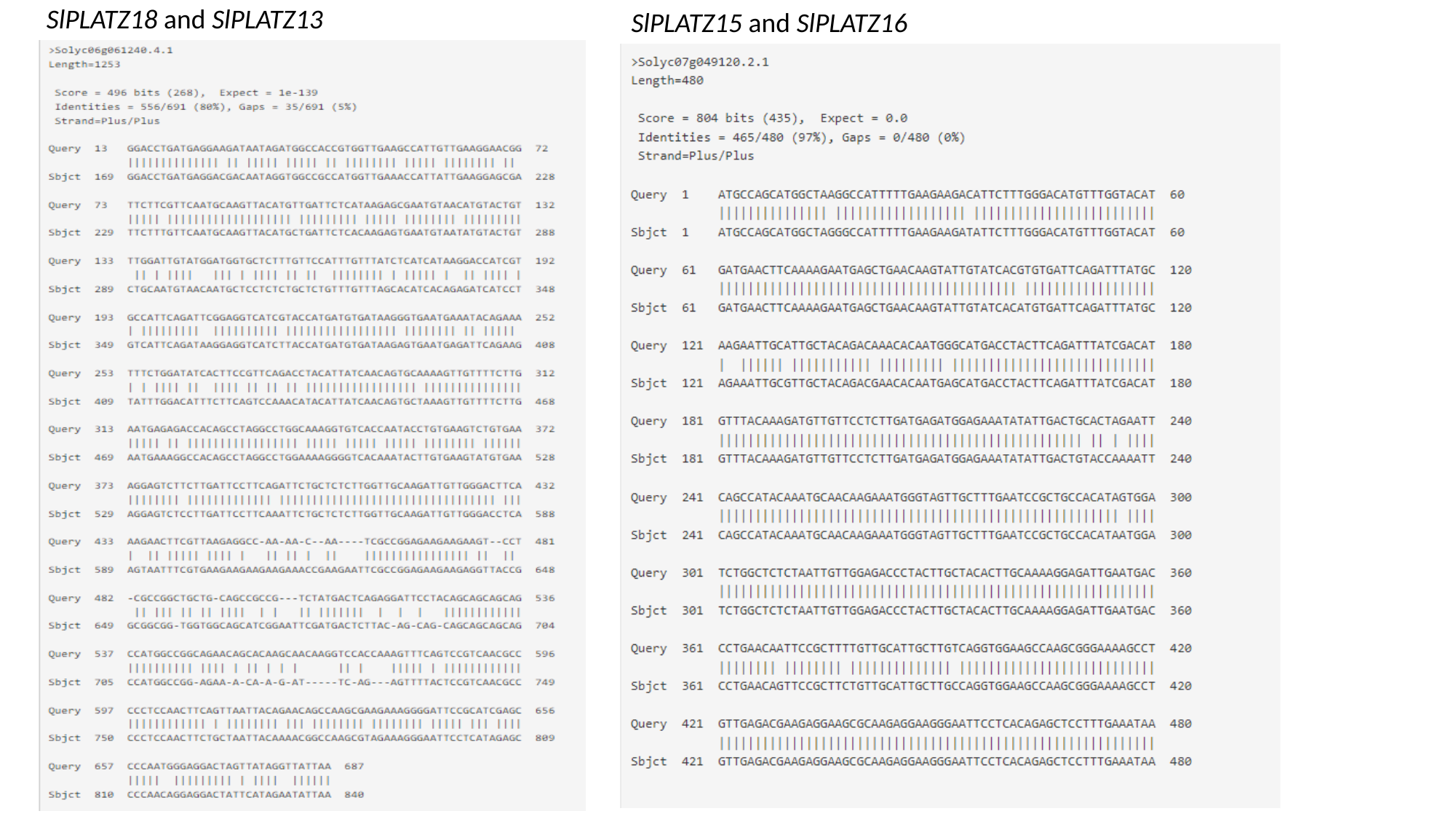

SlPLATZ15 and SlPLATZ16
SlPLATZ18 and SlPLATZ13

## Slide 4
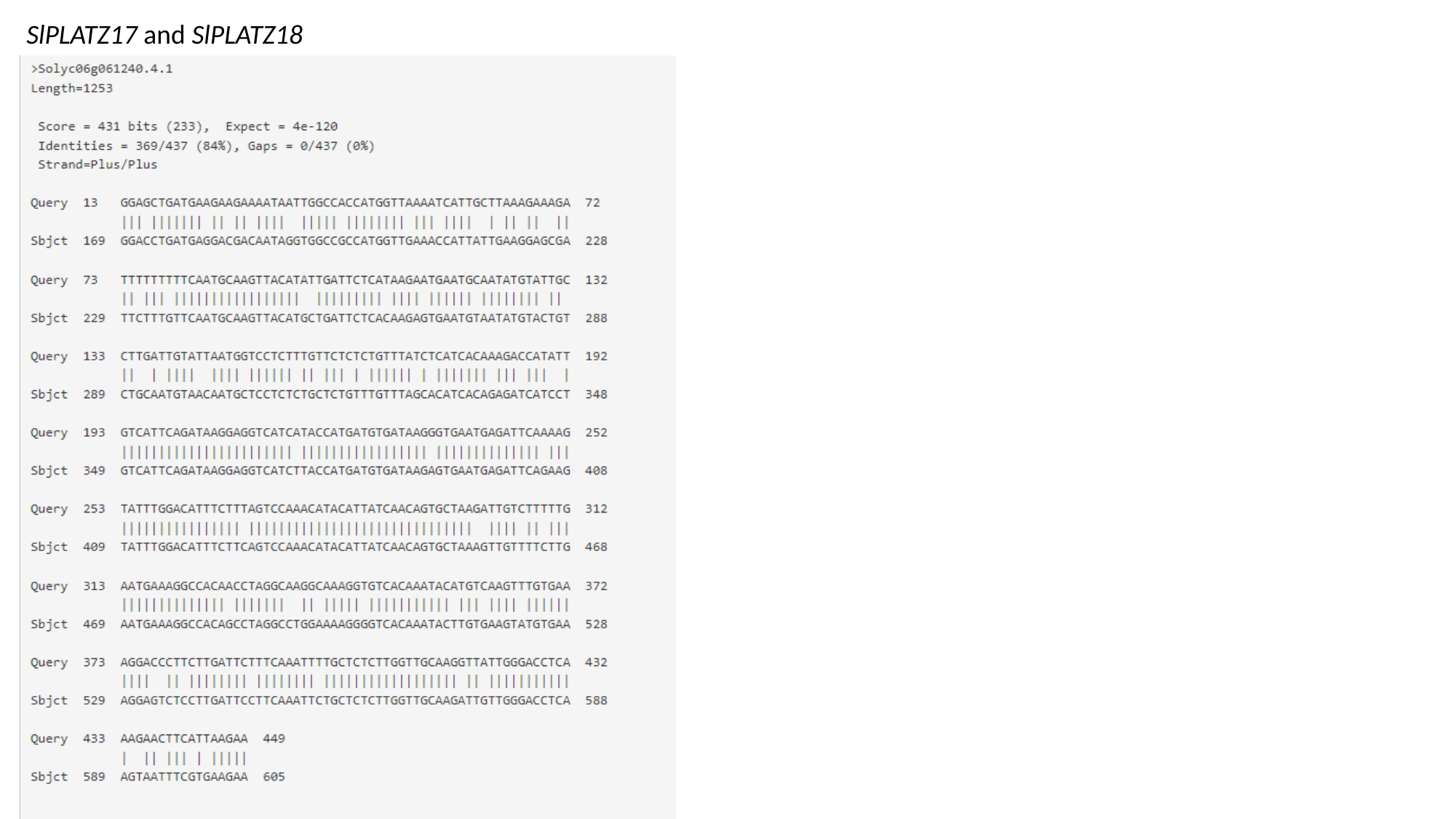

SlPLATZ17 and SlPLATZ18
