# Supplementary material for: Comprehensive Genome-Wide Analysis and Expression Pattern Profiling of PLATZ Gene Family Members in Solanum Lycopersicum L. under Multiple Abiotic Stresses
Source: Plants (Basel). 2022 Nov 15;11(22):3112. doi: 10.3390/plants11223112 (PMC9697139; doi:10.3390/plants11223112)
Supplement: Supplementary file 1 [file plants-11-03112-s001.zip › supplementary material.pdf]

Supplementary Materials: Comprehensive genome-wide analysis and expression pattern profiling of *PLATZ* gene family members in *Solanum lycopersicum* L. under multiple abiotic stresses

Antt Htet Wai, Md Mustafizur Rahman, Muhammad Waseem, Lae-Hyeon Cho, Aung Htay Naing, Jong-Seong Jeon, Do-jin Lee, Chang-Kil Kim and Mi-Young Chung*

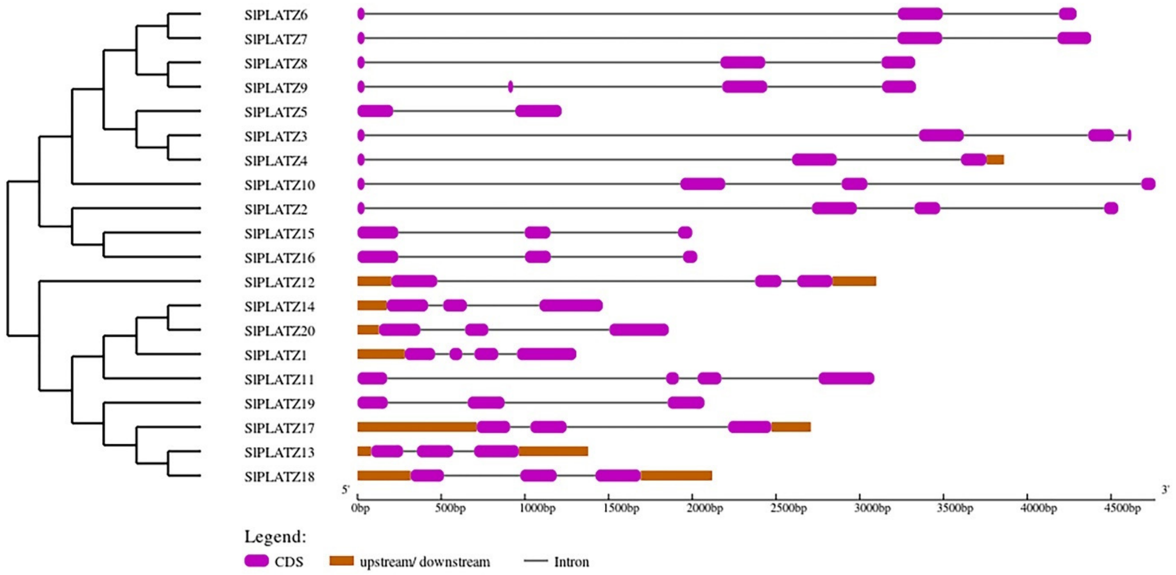

Figure S1. Schematic depiction of the exon-intron distribution of the *PLATZ* genes in tomato. Purple boxes indicate exons, black lines depict introns, and brown boxes represent untranslated regions.

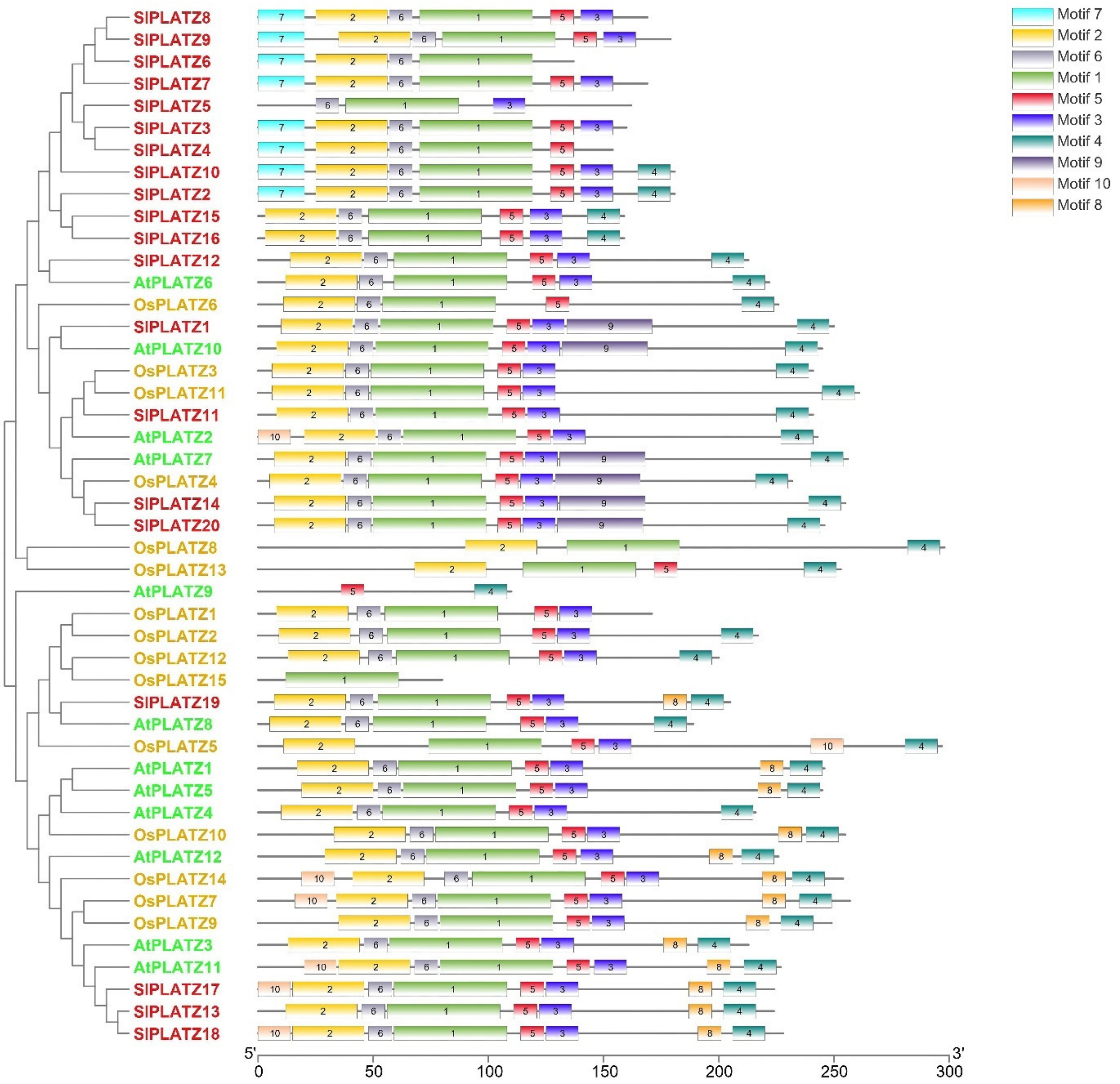

Figure S2. Schematic representation of 10 conserved motifs identified in the PLATZ proteins from tomato, Arabidopsis, and rice. The PLATZ domains from all three species harbor Motif 1. Different motifs are signified by different colored boxes, with the motif names described in the center.

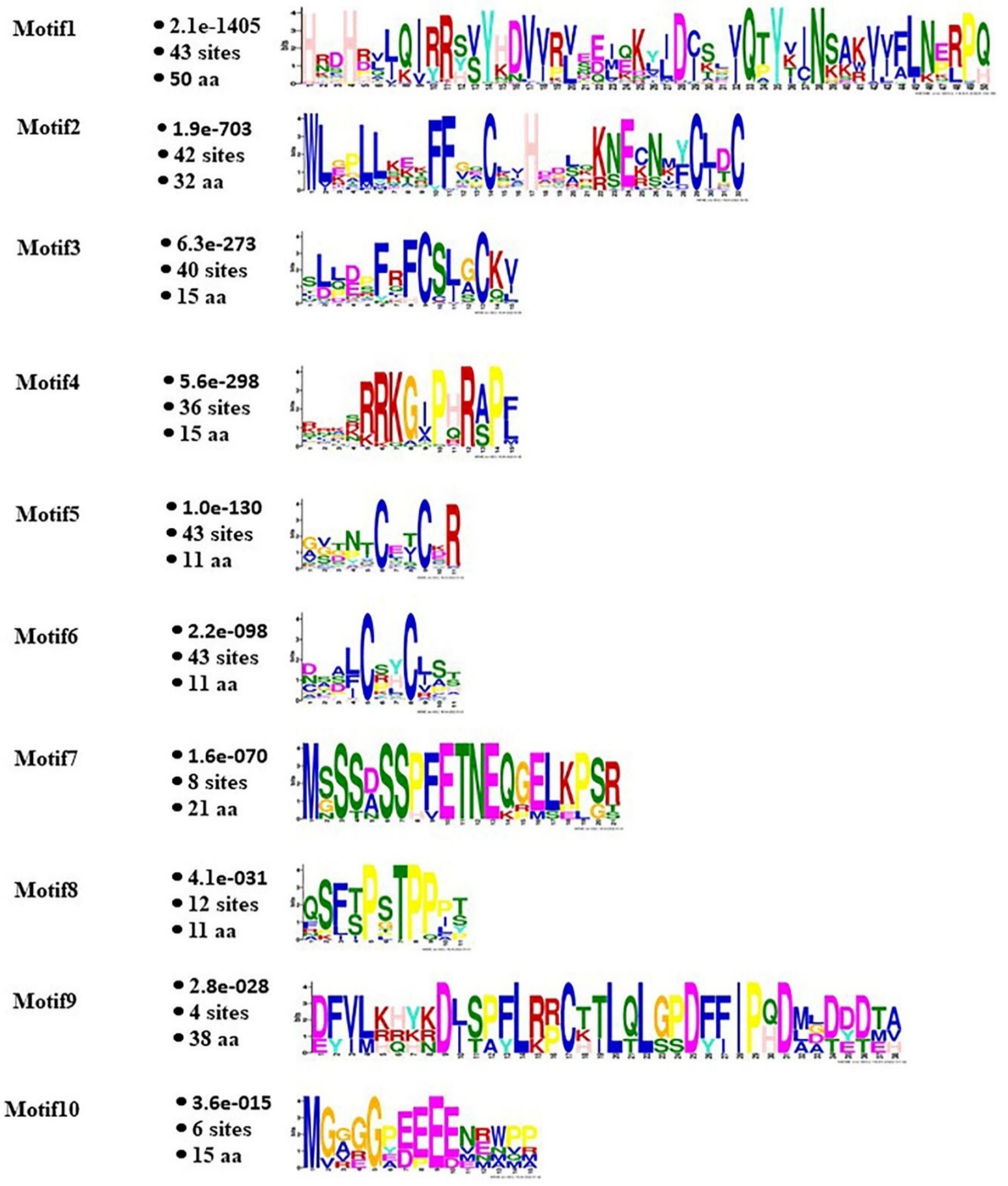

Figure S3. Overview of conserved motifs of PLATZ proteins from tomato, Arabidopsis and rice investigated by the MEME web tool.

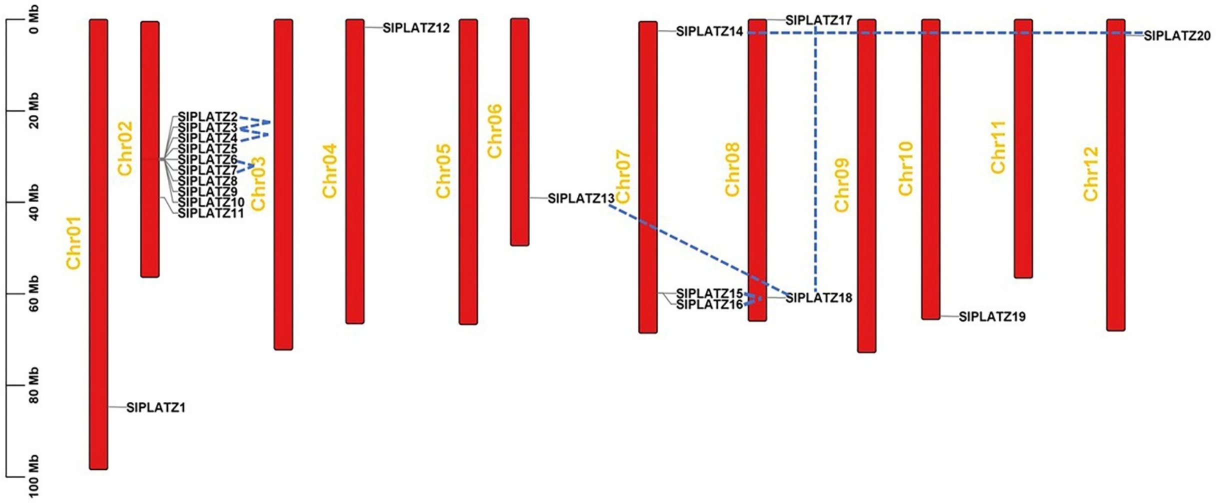

Figure S4. Chromosomal distribution of the *SlPLATZ* gene family. The chromosome numbers are indicated along with each chromosome. The scale bar in megabases (Mb) is drawn on the left of the figure. The duplicated gene pairs are connected by the blue dotted lines.

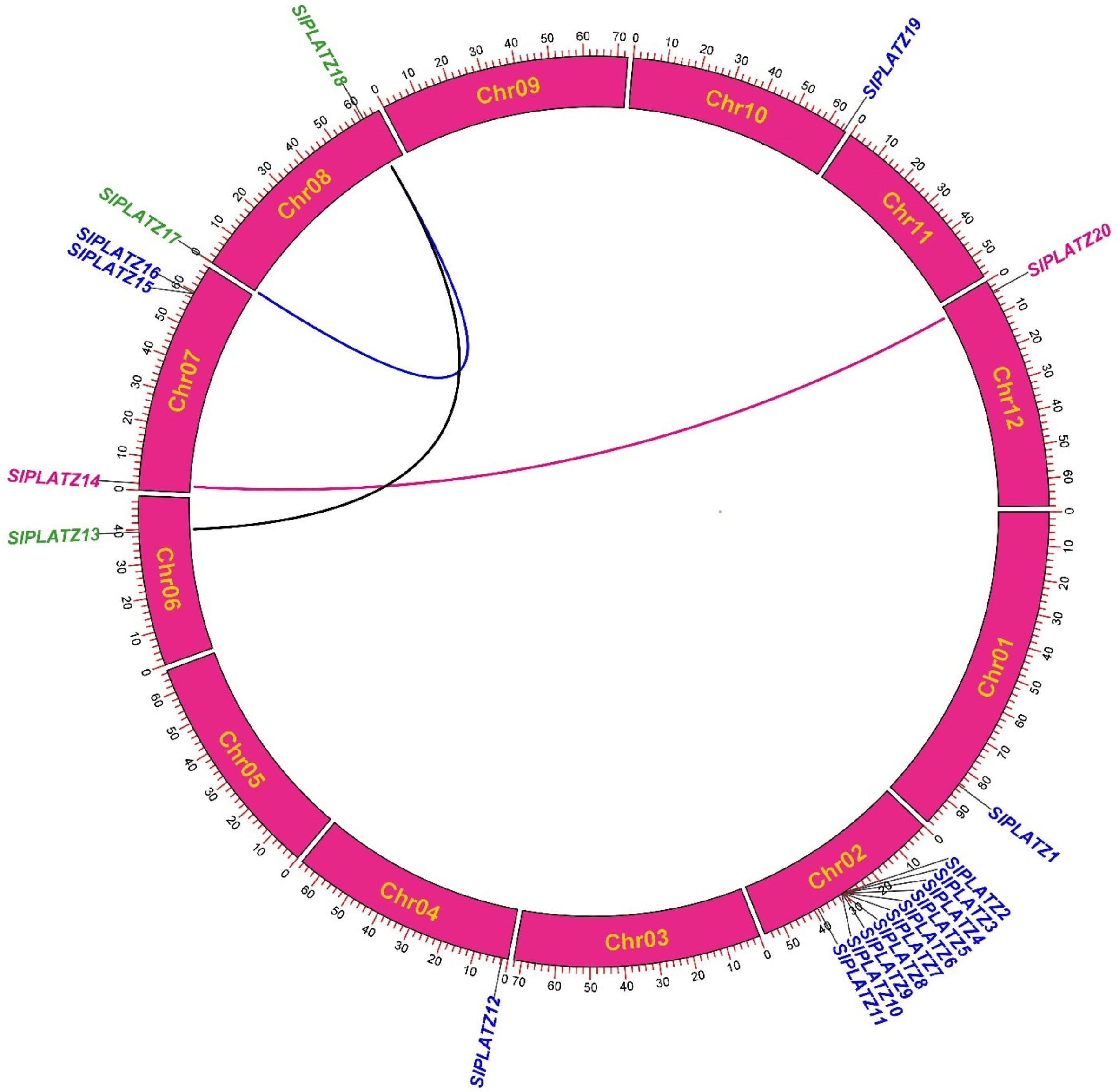

Figure S5. Schematic diagram of the segmentally duplicated *SlPLATZ* gene pairs identified in tomato. The duplicated gene pairs are linked by colored lines. Chromosome numbers and gene locations are depicted, and the chromosome size is indicated on a megabase scale (Mb).

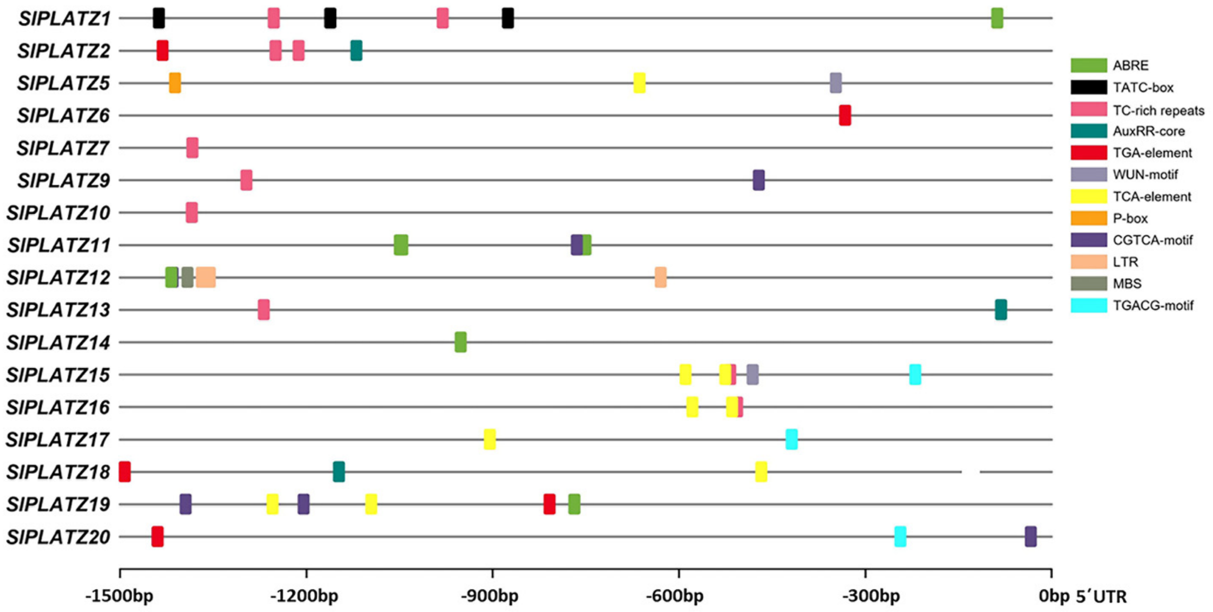

Figure. S6. Prediction of hormone and stress related *cis*-regulatory elements in the promoter regions of *SlPLATZ* genes. Elements identified are: a wound-responsive element (WUN-moti f), drought response related MYB-binding site (MBS), low-temperature-responsive element (LTR), P-box and TATC-elements implicated in GA response, defense and stress-responsive elements (TC-rich repeats), jasmonic acid-responsive elements (TGACG- and CGTCA-motif) , TCA-elements (related to the SA response), AuxRR-core and TGA-elements responsive to A uxin, and ABA-responsive elements (ABRE). The positions of *cis*-acting elements in regard to the start codon (taken as +1 bp) can be estimated using the scale below.

Table S1. List of *cis*-regulatory elements predicted in the promoters of tomato *PLATZ* genes.

Gene	Cis element	Consensus sequence	Location	Strand	organism	Function
SlPLATZ1	G-box	TACGTG	77	+	*Arabidopsis thaliana*	cis-acting regulatory element involved in light responsiveness
	ABRE	ACGTG	78	+	*Arabidopsis thaliana*	cis-acting element involved in the abscisic acid responsiveness
	GCN4_motif	TGAGTCA	114	+	*Oryza sativa*	cis-regulatory element involved in endosperm expression
	Box 4	ATTAAT	138	+	*Petroselinum crispum*	part of a conserved DNA module involved in light responsiveness
	TCT-motif	TCTTAC	163	-	*Arabidopsis thaliana*	part of a light responsive element
	LAMP-element	CTTTATCA	325	+	*Pisum sativum*	part of a light responsive element
	TATC-box	TATCCCA	866	+	*Oryza sativa*	cis-acting element involved in gibberellin-responsiveness
	Box 4	ATTAAT	922	-	*Petroselinum crispum*	part of a conserved DNA module involved in light responsiveness
	GT1-motif	GGTTAAT	940	-	*Avena sativa*	light responsive element
	TC-rich repeats	GTTTTCTTAC	971	+	*Nicotiana tabacum*	cis-acting element involved in defense and stress responsiveness
	ARE	AAACCA	1119	+	*Zea mays*	cis-acting regulatory element essential for the anaerobic induction
	TATC-box	TATCCCA	1152	-	*Oryza sativa*	cis-acting element involved in gibberellin-responsiveness
	Box 4	ATTAAT	1203	-	*Petroselinum crispum*	part of a conserved DNA module involved in light responsiveness
	ARE	AAACCA	1218	+	*Zea mays*	cis-acting regulatory element essential for the anaerobic induction
	TC-rich repeats	ATTCTCTAAC	1243	+	*Nicotiana tabacum*	cis-acting element involved in defense and stress responsiveness
	TATC-box	TATCCCA	1428	-	*Oryza sativa*	cis-acting element involved in gibberellin-responsiveness
SlPLATZ2	TCT-motif	TCTTAC	175	+	*Arabidopsis thaliana*	part of a light responsive element
	Box 4	ATTAAT	208	+	*Petroselinum crispum*	part of a conserved DNA module involved in light responsiveness
	MRE	AACCTAA	332	+	*Petroselinum crispum*	MYB binding site involved in light responsiveness
	GT1-motif	GGTTAAT	409	-	*Avena sativa*	light responsive element
	MBSI	aaaAaaC(G/C)GTTA	411	-	*Petunia hybrida*	MYB binding site involved in flavonoid biosynthetic genes regulation
	GT1-motif	GGTTAA	548	-	*Arabidopsis thaliana*	light responsive element

	Box 4	ATTAAT	761	-	*Petroselinum crispum*	part of a conserved DNA module involved in light responsiveness
	chs-CMA1a	TTACTTAA	794	-	*Daucus carota*	part of a light responsive element
	AE-box	AGAAACTT	908	+	*Arabidopsis thaliana*	part of a module for light response
	GA-motif	ATAGATAA	967	+	*Arabidopsis thaliana*	part of a light responsive element
	I-box	AGATAAGG	969	+	*Triticum aestivum*	part of a light responsive element
	AuxRR-core	GGTCCAT	1110	+	*Nicotiana tabacum*	cis-acting regulatory element involved in auxin responsiveness
	TCT-motif	TCTTAC	1134	-	*Arabidopsis thaliana*	part of a light responsive element
	TC-rich repeats	ATTCTCTAAC	1203	-	*Nicotiana tabacum*	cis-acting element involved in defense and stress responsiveness
	CCAAT-box	CAACGG	1220	-	*Hordeum vulgare*	MYBHv1 binding site
	TC-rich repeats	GTTTTCTTAC	1240	-	*Nicotiana tabacum*	cis-acting element involved in defense and stress responsiveness
	TCCC-motif	TCTCCCT	1389	-	*Spinacia oleracea*	part of a light responsive element
	TGA-element	AACGAC	1422	-	*Brassica oleracea*	auxin-responsive element
SlPLATZ3	GT1-motif	GGTTAA	157	+	*Arabidopsis thaliana*	light responsive element
	ARE	AAACCA	231	-	*Zea mays*	cis-acting regulatory element essential for the anaerobic induction
	CCAAT-box	CAACGG	323	-	*Hordeum vulgare*	MYBHv1 binding site
	TCT-motif	TCTTAC	353	+	*Arabidopsis thaliana*	part of a light responsive element
	Box 4	ATTAAT	386	+	*Petroselinum crispum*	part of a conserved DNA module involved in light responsiveness
	GT1-motif	GGTTAA	426	-	*Arabidopsis thaliana*	light responsive element
	GT1-motif	GGTTAA	549	+	*Arabidopsis thaliana*	light responsive element
	AE-box	AGAAACAA	964	+	*Arabidopsis thaliana*	part of a module for light response
	GA-motif	ATAGATAA	1037	+	*Arabidopsis thaliana*	part of a light responsive element
SlPLATZ4	GT1-motif	GGTTAA	161	+	*Arabidopsis thaliana*	light responsive element
	GT1-motif	GGTTAAT	321	-	*Avena sativa*	light responsive element
	CCAAT-box	CAACGG	327	-	*Hordeum vulgare*	MYBHv1 binding site
	TCT-motif	TCTTAC	357	+	*Arabidopsis thaliana*	part of a light responsive element
	Box 4	ATTAAT	390	+	*Petroselinum crispum*	part of a conserved DNA module involved in light responsiveness

	GT1-motif	GGTTAA	432	-	*Arabidopsis thaliana*	light responsive element
	GT1-motif	GGTTAA	555	+	*Arabidopsis thaliana*	light responsive element
	AE-box	AGAAACAA	969	+	*Arabidopsis thaliana*	part of a module for light response
	GA-motif	ATAGATAA	1042	+	*Arabidopsis thaliana*	part of a light responsive element
SlPLATZ5	ARE	AAACCA	8	+	*Zea mays*	cis-acting regulatory element essential for the anaerobic induction
	Box 4	ATTAAT	157	+	*Petroselinum crispum*	part of a conserved DNA module involved in light responsiveness
	GT1-motif	GGTTAAT	291	-	*Avena sativa*	light responsive element
	WUN-motif	AAATTTCCT	338	-	*Brassica oleracea*	wound-responsive element
	AE-box	AGAAACAA	441	-	*Arabidopsis thaliana*	part of a module for light response
	GATA-motif	AAGATAAGATT	517	+	*Arabidopsis thaliana*	part of a light responsive element
	TCA-element	CCATCTTTTT	654	+	*Nicotiana tabacum*	cis-acting element involved in salicylic acid responsiveness
	O2-site	GATGATGTGG	712	+	*Zea mays*	cis-acting regulatory element involved in zein metabolism regulation
	AE-box	AGAAACAA	924	-	*Arabidopsis thaliana*	part of a module for light response
	I-box	cCATATCCAAT	972	-	*Flaveria trinervia*	part of a light responsive element
	Box 4	ATTAAT	1029	-	*Petroselinum crispum*	part of a conserved DNA module involved in light responsiveness
	P-box	CCTTTTG	1402	+	*Oryza sativa*	gibberellin-responsive element
	HD-Zip 3	GTAAT(G/C)ATTAC	1450	-	*Arabidopsis thaliana*	protein binding site
SlPLATZ6	ARE	AAACCA	7	-	*Zea mays*	cis-acting regulatory element essential for the anaerobic induction
	TGA-element	AACGAC	323	-	*Brassica oleracea*	auxin-responsive element
	Box 4	ATTAAT	388	+	*Petroselinum crispum*	part of a conserved DNA module involved in light responsiveness
	GT1-motif	GGTTAA	427	-	*Arabidopsis thaliana*	light responsive element
	ARE	AAACCA	523	+	*Zea mays*	cis-acting regulatory element essential for the anaerobic induction
	ARE	AAACCA	1004	-	*Zea mays*	cis-acting regulatory element essential for the anaerobic induction
	sbp-CMA1c	CTTTATCTCTTCCA	1034	-	*Arabidopsis thaliana*	part of a light responsive element
	GA-motif	ATAGATAA	1038	+	*Arabidopsis thaliana*	part of a light responsive element
	AE-box	AGAAACAA	1263	-	*Arabidopsis thaliana*	part of a module for light response

SlPLATZ7	GT1-motif	GGTTAAT	350	-	*Avena sativa*	light responsive element
	CCAAT-box	CAACGG	356	-	*Hordeum vulgare*	MYBHv1 binding site
	TCT-motif	TCTTAC	386	+	*Arabidopsis thaliana*	part of a light responsive element
	Box 4	ATTAAT	419	+	*Petroselinum crispum*	part of a conserved DNA module involved in light responsiveness
	GT1-motif	GGTTAA	460	-	*Arabidopsis thaliana*	light responsive element
	ARE	AAACCA	556	+	*Zea mays*	cis-acting regulatory element essential for the anaerobic induction
	GT1-motif	GGTTAA	582	+	*Arabidopsis thaliana*	light responsive element
	GA-motif	ATAGATAA	1036	+	*Arabidopsis thaliana*	part of a light responsive element
	TC-rich repeats	GTTTTCTTAC	1374	+	*Nicotiana tabacum*	cis-acting element involved in defense and stress responsiveness
SlPLATZ8	CCAAT-box	CAACGG	353	-	*Hordeum vulgare*	MYBHv1 binding site
	Box 4	ATTAAT	416	+	*Petroselinum crispum*	part of a conserved DNA module involved in light responsiveness
	GT1-motif	GGTTAA	578	+	*Arabidopsis thaliana*	light responsive element
	TCT-motif	TCTTAC	383	+	*Arabidopsis thaliana*	part of a light responsive element
	GA-motif	ATAGATAA	1066	+	*Arabidopsis thaliana*	part of a light responsive element
SlPLATZ9	LTR	CCGAAA	87	-	*Hordeum vulgare*	cis-acting element involved in low-temperature responsiveness
	GT1-motif	GGTTAA	104	+	*Arabidopsis thaliana*	light responsive element
	ARE	AAACCA	178	+	*Zea mays*	cis-acting regulatory element essential for the anaerobic induction
	GT1-motif	GGTTAAT	304	+	*Avena sativa*	light responsive element
	Box 4	ATTAAT	309	+	*Petroselinum crispum*	part of a conserved DNA module involved in light responsiveness
	Box 4	ATTAAT	391	+	*Petroselinum crispum*	part of a conserved DNA module involved in light responsiveness
	Unnamed__1	GAATTTAATTAA	408	+	*Glycine max*	60K protein binding site
	Box 4	ATTAAT	441	+	*Petroselinum crispum*	part of a conserved DNA module involved in light responsiveness
	CGTCA-motif	CGTCA	462	-	*Hordeum vulgare*	cis-acting regulatory element involved in the MeJA-responsiveness
	Box 4	ATTAAT	498	+	*Petroselinum crispum*	part of a conserved DNA module involved in light responsiveness
	ARE	AAACCA	550	+	*Zea mays*	cis-acting regulatory element essential for the anaerobic induction
	ARE	AAACCA	708	+	*Zea mays*	cis-acting regulatory element essential for the anaerobic induction

	Box 4	ATTAAT	736	+	*Petroselinum crispum*	part of a conserved DNA module involved in light responsiveness
	GA-motif	ATAGATAA	1064	+	*Arabidopsis thaliana*	part of a light responsive element
	GT1-motif	GGTTAA	1213	+	*Arabidopsis thaliana*	light responsive element
	TC-rich repeats	ATTCTCTAAC	1287	-	*Nicotiana tabacum*	cis-acting element involved in defense and stress responsiveness
	chs-CMA2a	TCACTTGA	1399	-	*Petroselinum crispum*	part of a light responsive element
SlPLATZ10	GT1-motif	GGTTAAT	382	-	*Avena sativa*	light responsive element
	CCAAT-box	CAACGG	388	-	*Hordeum vulgare*	MYBHv1 binding site
	TCT-motif	TCTTAC	418	+	*Arabidopsis thaliana*	part of a light responsive element
	Box 4	ATTAAT	451	+	*Petroselinum crispum*	part of a conserved DNA module involved in light responsiveness
	GT1-motif	GGTTAA	494	-	*Arabidopsis thaliana*	light responsive element
	ARE	AAACCA	590	+	*Zea mays*	cis-acting regulatory element essential for the anaerobic induction
	GT1-motif	GGTTAAT	616	+	*Avena sativa*	light responsive element
	chs-CMA1a	TTACTTAA	673	+	*Daucus carota*	part of a light responsive element
	GA-motif	ATAGATAA	1065	+	*Arabidopsis thaliana*	part of a light responsive element
	TC-rich repeats	GTTTTCTTAC	1375	+	*Nicotiana tabacum*	cis-acting element involved in defense and stress responsiveness
	TCT-motif	TCTTAC	1379	+	*Arabidopsis thaliana*	part of a light responsive element
SlPLATZ11	ACE	CTAACGTATT	924	+	*Petroselinum crispum*	cis-acting element involved in light responsiveness
	ACE	GACACGTATG	1037	-	*Petroselinum crispum*	cis-acting element involved in light responsiveness
	chs-CMA1a	TTACTTAA	142	+	*Daucus carota*	part of a light responsive element
	Box 4	ATTAAT	163	+	*Petroselinum crispum*	part of a conserved DNA module involved in light responsiveness
	ARE	AAACCA	285	+	*Zea mays*	cis-acting regulatory element essential for the anaerobic induction
	ARE	AAACCA	315	-	*Zea mays*	cis-acting regulatory element essential for the anaerobic induction
	I-box	TGATAATGT	327	+	*Solanum tuberosum*	part of a light responsive element
	TCT-motif	TCTTAC	366	-	*Arabidopsis thaliana*	part of a light responsive element
	Box 4	ATTAAT	419	+	*Petroselinum crispum*	part of a conserved DNA module involved in light responsiveness
	AT-rich element	ATAGAAATCAA	537	+	*Glycine max*	binding site of AT-rich DNA binding protein (ATBP-1)

	Box 4	ATTAAT	543	+	*Petroselinum crispum*	part of a conserved DNA module involved in light responsiveness
	AT-rich element	ATAGAAATCAA	544	-	*Glycine max*	binding site of AT-rich DNA binding protein (ATBP-1)
	Box 4	ATTAAT	558	+	*Petroselinum crispum*	part of a conserved DNA module involved in light responsiveness
	Box 4	ATTAAT	622	+	*Petroselinum crispum*	part of a conserved DNA module involved in light responsiveness
	Box 4	ATTAAT	626	+	*Petroselinum crispum*	part of a conserved DNA module involved in light responsiveness
	G-Box	CACGTT	740	-	*Pisum sativum*	cis-acting regulatory element involved in light responsiveness
	ABRE	ACGTG	741	+	*Arabidopsis thaliana*	cis-acting element involved in the abscisic acid responsiveness
	TGACG-motif	TGACG	755	-	*Hordeum vulgare*	cis-acting regulatory element involved in the MeJA-responsiveness
	ATCT-motif	AATCTAATCC	781	-	*Pisum sativum*	part of a conserved DNA module involved in light responsiveness
	GT1-motif	GGTTAA	1033	-	*Arabidopsis thaliana*	light responsive element
	ABRE	GACACGTACGT	1036	-	*Oryza sativa*	cis-acting element involved in the abscisic acid responsiveness
	G-box	TAACACGTAG	1038	-	*Brassica oleracea*	cis-acting regulatory element involved in light responsiveness
	G-box	TACGTG	1039	+	*Arabidopsis thaliana*	cis-acting regulatory element involved in light responsiveness
	ABRE	TACGTGTC	1039	+	*Oryza sativa*	cis-acting element involved in the abscisic acid responsiveness
	ABRE	ACGTG	1040	+	*Arabidopsis thaliana*	cis-acting element involved in the abscisic acid responsiveness
	ARE	AAACCA	1162	+	*Zea mays*	cis-acting regulatory element essential for the anaerobic induction
	ARE	AAACCA	1269	+	*Zea mays*	cis-acting regulatory element essential for the anaerobic induction
SlPLATZ12	Box 4	ATTAAT	289	+	*Petroselinum crispum*	part of a conserved DNA module involved in light responsiveness
	Box 4	ATTAAT	342	+	*Petroselinum crispum*	part of a conserved DNA module involved in light responsiveness
	Box 4	ATTAAT	437	+	*Petroselinum crispum*	part of a conserved DNA module involved in light responsiveness
	GT1-motif	GGTTAA	466	-	*Arabidopsis thaliana*	light responsive element
	ARE	AAACCA	543	+	*Zea mays*	cis-acting regulatory element essential for the anaerobic induction
	ARE	AAACCA	573	+	*Zea mays*	cis-acting regulatory element essential for the anaerobic induction
	ARE	AAACCA	578	+	*Zea mays*	cis-acting regulatory element essential for the anaerobic induction
	LTR	CCGAAA	620	-	*Hordeum vulgare*	cis-acting element involved in low-temperature responsiveness
	GT1-motif	GGTTAAT	646	-	*Avena sativa*	light responsive element

	GT1-motif	GGTTAA	647	-	*Arabidopsis thaliana*	light responsive element
	Box 4	ATTAAT	675	+	*Petroselinum crispum*	part of a conserved DNA module involved in light responsiveness
	Box 4	ATTAAT	990	-	*Petroselinum crispum*	part of a conserved DNA module involved in light responsiveness
	ARE	AAACCA	1057	+	*Zea mays*	cis-acting regulatory element essential for the anaerobic induction
	TCCC-motif	TCTCCCT	1256	+	*Spinacia oleracea*	part of a light responsive element
	LTR	CCGAAA	1346	+	*Hordeum vulgare*	cis-acting element involved in low-temperature responsiveness
	LTR	CCGAAA	1359	+	*Hordeum vulgare*	cis-acting element involved in low-temperature responsiveness
	MBS	CAACTG	1382	-	*Arabidopsis thaliana*	MYB binding site involved in drought-inducibility
	MRE	AACCTAA	1389	+	*Petroselinum crispum*	MYB binding site involved in light responsiveness
	CGTCA-motif	CGTCA	1406	-	*Hordeum vulgare*	cis-acting regulatory element involved in the MeJA-responsiveness
	G-box	CACGTC	1407	-	*Zea mays*	cis-acting regulatory element involved in light responsiveness
	ABRE	ACGTG	1408	+	*Arabidopsis thaliana*	cis-acting element involved in the abscisic acid responsiveness
SlPLATZ13	AuxRR-core	GGTCCAT	72	-	*Nicotiana tabacum*	cis-acting regulatory element involved in auxin responsiveness
	Box 4	ATTAAT	631	+	*Petroselinum crispum*	part of a conserved DNA module involved in light responsiveness
	ARE	AAACCA	699	-	*Zea mays*	cis-acting regulatory element essential for the anaerobic induction
	chs-CMA1a	TTACTTAA	794	+	*Daucus carota*	part of a light responsive element
	Box 4	ATTAAT	867	-	*Petroselinum crispum*	part of a conserved DNA module involved in light responsiveness
	Box 4	ATTAAT	914	-	*Petroselinum crispum*	part of a conserved DNA module involved in light responsiveness
	Box 4	ATTAAT	1028	-	*Petroselinum crispum*	part of a conserved DNA module involved in light responsiveness
	AT1-motif	AATTATTTTTTATT	1181	-	*Solanum tuberosum*	part of a light responsive module
	Box 4	ATTAAT	1207	-	*Petroselinum crispum*	part of a conserved DNA module involved in light responsiveness
	chs-CMA1a	TTACTTAA	1255	-	*Daucus carota*	part of a light responsive element
	TCT-motif	TCTTAC	1259	-	*Arabidopsis thaliana*	part of a light responsive element
	TC-rich repeats	GTTTTCTTAC	1259	-	*Nicotiana tabacum*	cis-acting element involved in defense and stress responsiveness
	ARE	AAACCA	1356	+	*Zea mays*	cis-acting regulatory element essential for the anaerobic induction
SlPLATZ14	Box 4	ATTAAT	25	+	*Petroselinum crispum*	part of a conserved DNA module involved in light responsiveness

	G-Box	CACGTT	942	+	*Pisum sativum*	cis-acting regulatory element involved in light responsiveness
	ABRE	ACGTG	942	-	*Arabidopsis thaliana*	cis-acting element involved in the abscisic acid responsiveness
	G-box	CACGAC	1178	-	*Zea mays*	cis-acting regulatory element involved in light responsiveness
	chs-CMA1a	TTACTTAA	1212	+	*Daucus carota*	part of a light responsive element
	circadian	CAAAGATATC	1230	+	*Lycopersicon esculentum*	cis-acting regulatory element involved in circadian control
	3-AF1 binding site	TAAGAGAGGAA	1361	-	*Solanum tuberosum*	light responsive element
	ARE	AAACCA	1483	-	*Zea mays*	cis-acting regulatory element essential for the anaerobic induction
SlPLATZ15	TCCC-motif	TCTCCCT	115	-	*Spinacia oleracea*	part of a light responsive element
	CAT-box	GCCACT	142	-	*Arabidopsis thaliana*	cis-acting regulatory element related to meristem expression
	CGTCA-motif	CGTCA	210	+	*Hordeum vulgare*	cis-acting regulatory element involved in the MeJA-responsiveness
	MBSI	aaaAaaC(G/C)GTTA	260	-	*Petunia hybrida*	MYB binding site involved in flavonoid biosynthetic genes regulation
	ARE	AAACCA	343	+	*Zea mays*	cis-acting regulatory element essential for the anaerobic induction
	WUN-motif	AAATTTCCT	472	+	*Brassica oleracea*	wound-responsive element
	ARE	AAACCA	490	+	*Zea mays*	cis-acting regulatory element essential for the anaerobic induction
	TC-rich repeats	ATTCTCTAAC	508	-	*Nicotiana tabacum*	cis-acting element involved in defense and stress responsiveness
	TCA-element	CCATCTTTTT	516	+	*Nicotiana tabacum*	cis-acting element involved in salicylic acid responsiveness
	TCA-element	CCATCTTTTT	580	-	*Nicotiana tabacum*	cis-acting element involved in salicylic acid responsiveness
	GATA-motif	GATAGGA	687	-	*Arabidopsis thaliana*	part of a light responsive element
	TCCC-motif	TCTCCCT	809	-	*Spinacia oleracea*	part of a light responsive element
	AE-box	AGAAACTT	938	-	*Arabidopsis thaliana*	part of a module for light response
	ATCT-motif	AATCTAATCC	1101	-	*Pisum sativum*	part of a conserved DNA module involved in light responsiveness
	GA-motif	ATAGATAA	1306	+	*Arabidopsis thaliana*	part of a light responsive element
	MRE	AACCTAA	1314	-	*Petroselinum crispum*	MYB binding site involved in light responsiveness
	HD-Zip 3	GTAAT(G/C)ATTAC	1455	-	*Arabidopsis thaliana*	protein binding site
SlPLATZ16	TCCC-motif	TCTCCCT	103	-	*Spinacia oleracea*	part of a light responsive element
	CAT-box	GCCACT	130	-	*Arabidopsis thaliana*	cis-acting regulatory element related to meristem expression

	G-box	CACGAC	199	-	*Zea mays*	cis-acting regulatory element involved in light responsiveness
	TCT-motif	TCTTAC	247	+	*Arabidopsis thaliana*	part of a light responsive element
	ARE	AAACCA	333	+	*Zea mays*	cis-acting regulatory element essential for the anaerobic induction
	TC-rich repeats	ATTCTCTAAC	497	-	*Nicotiana tabacum*	cis-acting element involved in defense and stress responsiveness
	TCA-element	CCATCTTTTT	505	+	*Nicotiana tabacum*	cis-acting element involved in salicylic acid responsiveness
	GATA-motif	GATAGGA	526	-	*Arabidopsis thaliana*	part of a light responsive element
	TCA-element	CCATCTTTTT	569	-	*Nicotiana tabacum*	cis-acting element involved in salicylic acid responsiveness
	GATA-motif	GATAGGA	676	-	*Arabidopsis thaliana*	part of a light responsive element
	TCCC-motif	TCTCCCT	799	-	*Spinacia oleracea*	part of a light responsive element
	AE-box	AGAAACTT	928	-	*Arabidopsis thaliana*	part of a module for light response
	ATCT-motif	AATCTAATCC	1091	-	*Pisum sativum*	part of a conserved DNA module involved in light responsiveness
	GA-motif	ATAGATAA	1306	+	*Arabidopsis thaliana*	part of a light responsive element
	MRE	AACCTAA	1314	-	*Petroselinum crispum*	MYB binding site involved in light responsiveness
	Box 4	ATTAAT	1449	-	*Petroselinum crispum*	part of a conserved DNA module involved in light responsiveness
SlPLATZ17	GT1-motif	GGTTAAT	15	+	*Avena sativa*	light responsive element
	GATA-motif	AAGATAAGATT	19	+	*Arabidopsis thaliana*	part of a light responsive element
	Box 4	ATTAAT	61	+	*Petroselinum crispum*	part of a conserved DNA module involved in light responsiveness
	GT1-motif	GGTTAAT	95	+	*Avena sativa*	light responsive element
	GATA-motif	AAGATAAGATT	99	+	*Arabidopsis thaliana*	part of a light responsive element
	Box III	atCATTTTCACt	369	-	*Pisum sativum*	protein binding site
	GA-motif	ATAGATAA	379	+	*Arabidopsis thaliana*	part of a light responsive element
	TGACG-motif	TGACG	409	+	*Hordeum vulgare*	cis-acting regulatory element involved in the MeJA-responsiveness
	Box 4	ATTAAT	662	+	*Petroselinum crispum*	part of a conserved DNA module involved in light responsiveness
	GATA-motif	GATAGGG	722	+	*Pisum sativum*	part of a light responsive element
	ARE	AAACCA	804	+	*Zea mays*	cis-acting regulatory element essential for the anaerobic induction
	GATA-motif	GATAGGA	879	+	*Arabidopsis thaliana*	part of a light responsive element

	TCA-element	CCATCTTTTT	895	-	*Nicotiana tabacum*	cis-acting element involved in salicylic acid responsiveness
SlPLATZ18	Box 4	ATTAAT	127	+	*Petroselinum crispum*	part of a conserved DNA module involved in light responsiveness
	ARE	AAACCA	139	-	*Zea mays*	cis-acting regulatory element essential for the anaerobic induction
	Box 4	ATTAAT	225	+	*Petroselinum crispum*	part of a conserved DNA module involved in light responsiveness
	Box 4	ATTAAT	403	+	*Petroselinum crispum*	part of a conserved DNA module involved in light responsiveness
	TCA-element	CCATCTTTTT	458	-	*Nicotiana tabacum*	cis-acting element involved in salicylic acid responsiveness
	GCN4_motif	TGAGTCA	534	-	*Oryza sativa*	cis-regulatory element involved in endosperm expression
	ARE	AAACCA	599	-	*Zea mays*	cis-acting regulatory element essential for the anaerobic induction
	Box 4	ATTAAT	817	-	*Petroselinum crispum*	part of a conserved DNA module involved in light responsiveness
	AE-box	AGAAACTT	835	-	*Arabidopsis thaliana*	part of a module for light response
	Box 4	ATTAAT	1003	-	*Petroselinum crispum*	part of a conserved DNA module involved in light responsiveness
	AuxRR-core	GGTCCAT	1138	-	*Nicotiana tabacum*	cis-acting regulatory element involved in auxin responsiveness
	Box 4	ATTAAT	1249	-	*Petroselinum crispum*	part of a conserved DNA module involved in light responsiveness
	MRE	AACCTAA	1422	-	*Petroselinum crispum*	MYB binding site involved in light responsiveness
	GATA-motif	AAGGATAAGG	1474	+	*Solanum tuberosum*	part of a light responsive element
	TGA-element	AACGAC	1483	+	*Brassica oleracea*	auxin-responsive element
SlPLATZ19	TATC-box	TATCCCA	168	+	*Oryza sativa*	cis-acting element involved in gibberellin-responsiveness
	ABRE	ACGTG	268	-	*Arabidopsis thaliana*	cis-acting element involved in the abscisic acid responsiveness
	G-box	TACGTG	268	-	*Arabidopsis thaliana*	cis-acting regulatory element involved in light responsiveness
	LTR	CCGAAA	456	+	*Hordeum vulgare*	cis-acting element involved in low-temperature responsiveness
	O2-site	GTTGACGTGA	465	+	*Zea mays*	cis-acting regulatory element involved in zein metabolism regulation
	G-Box	CACGTT	521	+	*Pisum sativum*	cis-acting regulatory element involved in light responsiveness
	ABRE	ACGTG	521	-	*Arabidopsis thaliana*	cis-acting element involved in the abscisic acid responsiveness
	TGA-element	AACGAC	610	+	*Brassica oleracea*	auxin-responsive element
	TCA-element	CCATCTTTTT	616	-	*Nicotiana tabacum*	cis-acting element involved in salicylic acid responsiveness
	Box II	TGGTAATAA	623	+	*Solanum tuberosum*	part of a light responsive element

	ARE	AAACCA	710	+	*Zea mays*	cis-acting regulatory element essential for the anaerobic induction
	O2-site	GATGATGTGG	732	-	*Zea mays*	cis-acting regulatory element involved in zein metabolism regulation
	G-box	ACACGTGT	758	-	*Brassica napus*	cis-acting regulatory element involved in light responsiveness
	ABRE	CACGTG	759	-	*Arabidopsis thaliana*	cis-acting element involved in the abscisic acid responsiveness
	GT1-motif	GGTTAA	790	-	*Arabidopsis thaliana*	light responsive element
	TGA-element	AACGAC	799	-	*Brassica oleracea*	auxin-responsive element
	Unnamed__1	GAATTTAATTAA	1026	+	*Glycine max*	60K protein binding site
	TCA-element	CCATCTTTTT	1086	-	*Nicotiana tabacum*	cis-acting element involved in salicylic acid responsiveness
	CCAAT-box	CAACGG	1151	+	*Hordeum vulgare*	MYBHv1 binding site
	CGTCA-motif	CGTCA	1195	+	*Hordeum vulgare*	cis-acting regulatory element involved in the MeJA-responsiveness
	TCA-element	CCATCTTTTT	1245	+	*Nicotiana tabacum*	cis-acting element involved in salicylic acid responsiveness
	MRE	AACCTAA	1282	-	*Petroselinum crispum*	MYB binding site involved in light responsiveness
	CGTCA-motif	CGTCA	1385	+	*Hordeum vulgare*	cis-acting regulatory element involved in the MeJA-responsiveness
	Box 4	ATTAAT	1436	-	*Petroselinum crispum*	part of a conserved DNA module involved in light responsiveness
SlPLATZ20	CGTCA-motif	CGTCA	24	-	*Hordeum vulgare*	cis-acting regulatory element involved in the MeJA-responsiveness
	TGACG-motif	TGACG	24	+	*Hordeum vulgare*	cis-acting regulatory element involved in the MeJA-responsiveness
	CGTCA-motif	CGTCA	234	+	*Hordeum vulgare*	cis-acting regulatory element involved in the MeJA-responsiveness
	AE-box	AGAAACAA	452	-	*Arabidopsis thaliana*	part of a module for light response
	Box 4	ATTAAT	484	+	*Petroselinum crispum*	part of a conserved DNA module involved in light responsiveness
	Box 4	ATTAAT	488	+	*Petroselinum crispum*	part of a conserved DNA module involved in light responsiveness
	Box 4	ATTAAT	500	+	*Petroselinum crispum*	part of a conserved DNA module involved in light responsiveness
	TCT-motif	TCTTAC	509	+	*Arabidopsis thaliana*	part of a light responsive element
	3-AF1 binding site	TAAGAGAGGAA	534	-	*Solanum tuberosum*	light responsive element
	Box 4	ATTAAT	560	+	*Petroselinum crispum*	part of a conserved DNA module involved in light responsiveness
	GATA-motif	AAGGATAAGG	924	+	*Solanum tuberosum*	part of a light responsive element
	TCT-motif	TCTTAC	990	+	*Arabidopsis thaliana*	part of a light responsive element

AT-rich sequence	TAAAATACT	1025	+	*Pisum sativum*	element for maximal elicitor-mediated activation (2copies)
Box 4	ATTAAT	1069	-	*Petroselinum crispum*	part of a conserved DNA module involved in light responsiveness
I-box	AGATAAGG	1105	-	*Triticum aestivum*	part of a light responsive element
Box 4	ATTAAT	1282	-	*Petroselinum crispum*	part of a conserved DNA module involved in light responsiveness
Box 4	ATTAAT	1286	-	*Petroselinum crispum*	part of a conserved DNA module involved in light responsiveness
TGA-element	AACGAC	1430	+	*Brassica oleracea*	auxin-responsive element

Table S2. miRNA Sequence characteristics of *PLATZ* genes in tomato.

miRNA_Acc.	Target gene	Expectation	miRNA_start	miRNA_end	Target_start	Target_end	miRNA_aligned_fragment	alignment	Target_aligned_fragment	Inhibition
sly-miR9469-3p	*SlPLATZ2*	5	1	21	403	423	AUUCGGUCUUCUUAUGUGGAC	...:: :::::::..: ::	ACUUGCAAAAGAAGAUUGCAU	Cleavage
sly-miR5303	*SlPLATZ5*	5	1	21	1	21	UUUUUGAAGAGUUCGAGCAAC	: ::: :.:::.:: ::.::	AUGGCUAGGACUUUUGAAGAA	Cleavage
sly-miR171a	*SlPLATZ6*	5	1	21	359	379	UGAUUGAGCCGUGCCAAUAUC	: ::::. .:::::: :::	AUUCUUGGUCUGGCUCAUUCA	Cleavage
sly-miR6024	*SlPLATZ11*	4.5	1	22	95	116	UUUUAGCAAGAGUUGUUUUACC	: :::::: ::.:::.: :.:	AGAAAAACAUCUUUUGUUUAGA	Cleavage
sly-miR162	*SlPLATZ11*	5	1	21	557	577	UCGAUAAACCUCUGCAUCCAG	: ::::::::::: :::	UAGAAUGCAGAGGUUCAUCUU	Cleavage
sly-miR9472-3p	*SlPLATZ11*	5	1	21	49	69	UUCACAAUCUCUGCUGAAAAA	:::: ..:::. ::::::	AAUUUCUUUGGAGGAUGUGAA	Cleavage
sly-miR9478-3p	*SlPLATZ12*	5	1	21	596	616	UUCGAUGACAUAUUUGAGCCU	.:...:::. ::::::..:	CUCUUUGAAUGAGUCAUCGGA	Translation
sly-miR319b	*SlPLATZ13*	3	1	21	247	267	UUGGACUGAAGGGAGCUCCCU	::: :..:::::::::::	UUGGACAUUUCUUCAGUCCAA	Cleavage
sly-miR319c-3p	*SlPLATZ13*	3	1	21	247	267	UUGGACUGAAGGGAGCUCCUU	::: :..:::::::::::	UUGGACAUUUCUUCAGUCCAA	Cleavage
sly-miR159	*SlPLATZ13*	3.5	1	21	248	268	UUUGGAUUGAAGGGAGCUCUA	:.::: :..:::::.::::::	UGGACAUUUCUUCAGUCCAAA	Cleavage
sly-miR319a	*SlPLATZ13*	4	1	20	249	268	CUUGGACUGAAGGGAGCUCC	::: :..:::::::::::	GGACAUUUCUUCAGUCCAAA	Cleavage
sly-miR9469-3p	*SlPLATZ15*	4	1	21	337	357	AUUCGGUCUUCUUAUGUGGAC	...:: :::.:::..::::	ACUUGCAAAAGGAGAUUGAAU	Cleavage
sly-miR403-5p	*SlPLATZ15*	5	1	21	107	127	CGUUUGUGCGUGAAUCUAACA	: :::::.:.::: :::.	AUUCAGAUUUAUGCAGAAAUU	Cleavage
sly-miR9469-3p	*SlPLATZ16*	4	1	21	337	357	AUUCGGUCUUCUUAUGUGGAC	...:: :::.:::..::::	ACUUGCAAAAGGAGAUUGAAU	Cleavage
sly-miR319b	*SlPLATZ17*	3.5	1	21	256	276	UUGGACUGAAGGGAGCUCCCU	::: :..:::.::::::::	UUGGACAUUUCUUUAGUCCAA	Cleavage
sly-miR319c-3p	*SlPLATZ17*	3.5	1	21	256	276	UUGGACUGAAGGGAGCUCCUU	::: :..:::.::::::::	UUGGACAUUUCUUUAGUCCAA	Cleavage
sly-miR159	*SlPLATZ17*	4	1	21	257	277	UUUGGAUUGAAGGGAGCUCUA	:.::: :..:::.:.::::::	UGGACAUUUCUUUAGUCCAAA	Cleavage
sly-miR319a	*SlPLATZ17*	4.5	1	20	258	277	CUUGGACUGAAGGGAGCUCC	::: :..:::.::::::::	GGACAUUUCUUUAGUCCAAA	Cleavage
sly-miR482a	*SlPLATZ18*	4.5	1	22	1111	1132	UUUCCAAUUCCACCCAUUCCUA	:.: :::::::: : ::::	GUUGGAGGGGUGGAAGUUGAAA	Cleavage
sly-miR396a-5p	*SlPLATZ19*	5	1	21	335	355	UUCCACAGCUUUCUUGAACUG	::::.. :::: :::::::	AUGUUUGUGAAAUAUGUGGAA	Cleavage
sly-miR396b	*SlPLATZ19*	5	1	21	335	355	UUCCACAGCUUUCUUGAACUU	: ::::.. :::: :::::::	AUGUUUGUGAAAUAUGUGGAA	Cleavage
sly-miR5303	*SlPLATZ20*	4.5	1	21	337	357	UUUUUGAAGAGUUCGAGCAAC	:: : :.: ::::::::.::	UGUGAUAGGAGUCUUCAAGAA	Translation

Table S3. Parameters used in 3D modeling of SlPLATZ proteins.

No.	Type	C-Score	No. of Decoys	Cluster Density
1	SlPLATZ1	-3.13	703	0.0507
2	SlPLATZ2	-3.77	635	0.0252
3	SlPLATZ3	-4.79	752	0.0092
4	SlPLATZ4	-4.25	1421	0.0161
5	SlPLATZ5	-4.17	1014	0.0179
6	SlPLATZ6	-3.96	1708	0.0199
7	SlPLATZ7	-4.84	768	0.0089
8	SlPLATZ8	-4.74	850	0.0097
9	SlPLATZ9	-4.75	734	0.0096
10	SlPLATZ10	-3.70	633	0.0270
11	SlPLATZ11	-3.55	667	0.0337
12	SlPLATZ12	-4.12	629	0.0173
13	SlPLATZ13	-3.62	707	0.0303
14	SlPLATZ14	-3.23	671	0.0436
15	SlPLATZ15	-4.30	1232	0.0145
16	SlPLATZ16	-4.42	1012	0.0127
17	SlPLATZ17	-4.11	663	0.0188
18	SlPLATZ18	-4.15	691	0.0186
19	SlPLATZ19	-4.49	904	0.0127
20	SlPLATZ20	-3.79	681	0.0253

C-Score, confident score.

Table S4. Model validation data for the predicted structures of SlPLATZ proteins.

No.	Locus name	Type	TM-Score	RMSD
1	Solyc01g091000	SlPLATZ1	0.36±0.12	13.3±4.1
2	Solyc02g033100	SlPLATZ2	0.31±0.10	14.1±3.8
3	Solyc02g033110	SlPLATZ3	0.22±0.06	16.6±2.9
4	Solyc02g033120	SlPLATZ4	0.27±0.08	15.0±3.5
5	Solyc02g036120	SlPLATZ5	0.27±0.08	14.9±3.6
6	Solyc02g036130	SlPLATZ6	0.29±0.09	13.9±3.9
7	Solyc02g036140	SlPLATZ7	0.22±0.06	16.9±2.8
8	Solyc02g036170	SlPLATZ8	0.23±0.06	16.6±2.9
9	Solyc02g036200	SlPLATZ9	0.23±0.06	16.8±2.8
10	Solyc02g036230	SlPLATZ10	0.31±0.10	13.9±3.9
11	Solyc02g068510	SlPLATZ11	0.32±0.11	14.3±3.8
12	Solyc04g008090	SlPLATZ12	0.28±0.09	15.5±3.3
13	Solyc06g061240	SlPLATZ13	0.32±0.11	14.3±3.8
14	Solyc07g007320	SlPLATZ14	0.35±0.12	13.6±4.0
15	Solyc07g049120	SlPLATZ15	0.26±0.08	15.2±3.5
16	Solyc07g049130	SlPLATZ16	0.25±0.07	15.5±3.3
17	Solyc08g005100	SlPLATZ17	0.28±0.09	15.6±3.3
18	Solyc08g076860	SlPLATZ18	0.27±0.08	15.8±3.2
19	Solyc10g085800	SlPLATZ19	0.25±0.07	16.5±3.0
20	Solyc12g010470	SlPLATZ20	0.30±0.10	15.0±3.5

TM-score, Template modeling score; RMSD, root-mean-square deviation between residues structurally aligned by TM-align.

Table S5. Elements of secondary structure predicted in SlPLATZ proteins.

No.	Protein name	α- Helix	Β-strand	Coil
1	SlPLATZ1	6	8	14
2	SlPLATZ2	4	7	11
3	SlPLATZ3	4	8	12
4	SlPLATZ4	4	5	10
5	SlPLATZ5	5	8	13
6	SlPLATZ6	2	6	8
7	SlPLATZ7	3	8	11
8	SlPLATZ8	4	8	12
9	SlPLATZ9	4	8	12
10	SlPLATZ10	5	6	11
11	SlPLATZ11	8	7	15
12	SlPLATZ12	2	7	9
13	SlPLATZ13	6	6	12
14	SlPLATZ14	7	7	14
15	SlPLATZ15	5	7	12
16	SlPLATZ16	4	7	11
17	SlPLATZ17	5	6	11
18	SlPLATZ18	5	7	10
19	SlPLATZ19	4	7	11
20	SlPLATZ20	6	7	13

Table S6. Predicted ligands and ligand-binding residues in SlPLATZ proteins.

No.	Protein name	Ligand name	Ligand binding site residue
1	SlPLATZ1	Nucleic acid	102,110,145,146,147,246,247,248,250
		Magnesium	130,131,216
2	SlPLATZ2	Nucleic acid	41,43,45,103,148,152
		Peptide	58,60,62,79
		Calium	38,116,159
3	SlPLATZ3	Zinc	54,57,71,74
4	SlPLATZ4	Zinc	39,42,62,65
		Glycine	75,76,88,90,96,98,99
		Calium	82,86,88,106,109
5	SlPLATZ5	Nucleic acid	68,93,96,97
		Magnesium	50,84,116
6	SlPLATZ6	Zinc	39,42,62,65
		Peptide	82,85,87
7	SlPLATZ7	Zinc	54,57,71,74
		Copper	62,65
8	SlPLATZ8	Chlorophyll A	26,27,30
		Alpha-D-Mannose	22,85
		Magnesium	30,148
9	SlPLATZ9	Zinc	49,52,72,75
10	SlPLATZ10	Peptide	87,97,99,101,102,103,113,114,115,116,117
		Calium	39,157

11	SlPLATZ11	Alpha-D-Mannose	34,35,87,88,90,92,93
		Glutathione	32,42,45,89
12	SlPLATZ12	Copper	51,54
		Phosphate	96,144,153
13	SlPLATZ13	Nucleic acid	112,115,116,117,218,220,222
		Magnesium	100,101,191
14	SlPLATZ14	Beta-D-glucose	70,84
		Magnesium	4,5,19,20,34,98
15	SlPLATZ15	Zinc	17,20,40,43
16	SlPLATZ16	Zinc	32,35,49,52
17	SlPLATZ17	Nucleic acid	107,108,111,112
		Beta-D-Mannose	107,108
		Calium	42,97,99
18	SlPLATZ18	Nucleic acid	48,49,50,54,57,61,65,66,68,70,71,95,96,97,98,99,126
		Zinc	53,56
19	SlPLATZ19	Zinc	31,35,82
		Cyanide	34,35,127
		Calium	33,37
20	SlPLATZ20	Magnesium	71,72
		Copper	61,65

Table S7. Gene Ontology (GO) annotation for SlPLATZ proteins.

Name	Molecular Process	Biological Process	Cellular component
SlPLATZ1	structural constituent of ribosome	translation	cytosolic ribosome, large ribosomal subunit
SlPLATZ2	structural molecule activity	NA	virion component
SlPLATZ3	metal ion binding, heme oxygenase (decyclizing) activity, pyrroloquinoline-quinone synthase activity	heme oxidation, pyrroloquinoline quinone biosynthetic process	extracellular region, periplasmic space
SlPLATZ4	amino acid binding, metal ion binding, aminopeptidase activity	glutamine metabolic process, protein maturation, asparagine biosynthetic process	Cytoplasm, extracellular region,
SlPLATZ5	purine ribonucleoside triphosphate binding, adenyl ribonucleotide binding, pyrophosphatase activity	regulation of DNA replication, DNA replication initiation, regulation of transcription	cytoplasm
SlPLATZ6	choloylglycine hydrolase activity, isopenicillin-N N-acyltransferase activity, metalloendopeptidase activity	choloylglycine hydrolase activity, hydrolase activity	NA
SlPLATZ7	ion binding	cellular defense response, phospholipid catabolic process, histidine catabolic process	extracellular space
SlPLATZ8	oxidoreductase activity, acting on the CH-NH group of donors, NAD or NADP as acceptor	nucleotide metabolic process, nucleobase-containing compound biosynthetic process, glycine metabolic process	Cytoplasm, viral capsid
SlPLATZ9	ion binding	inflammatory response, antibiotic biosynthetic process, glycolytic process	Cytoplasm, extracellular space

SlPLATZ10	RNA binding, structural molecule activity	NA	virion component
SlPLATZ11	interleukin-1, type I, activating receptor activity, Copper binding, oxidoreductase activity	cell adhesion, obsolete oxidation-reduction process	membrane
SlPLATZ12	protein binding, molybdenum ion binding, electron transfer activity	obsolete oxidation-reduction process, antigen processing and presentation of endogenous peptide antigen via MHC class I	mitochondrial intermembrane space, membrane
SlPLATZ13	nucleotide binding, obsolete transcription regulator activity, protein binding, rRNA binding	gene expression, cellular macromolecule biosynthetic process, protein metabolic process	protein-containing complex, intracellular non-membrane-bounded organelle, obsolete cytoplasmic part
SlPLATZ14	ion binding, protein binding	obsolete oxidation-reduction process, cell adhesion, antibiotic biosynthetic process	NA
SlPLATZ15	glutamate dehydrogenase [NAD(P)+] activity, metal ion binding, NAD binding,	obsolete oxidation-reduction process, cellular amino acid metabolic process	mitochondrial matrix, integral component of membrane
SlPLATZ16	ion binding	glycolytic process, fatty acid biosynthetic process, proteolysis	phosphopyruvate hydratase complex, plasma membrane
SlPLATZ17	structural constituent of ribosome, ATP binding, chaperone binding, large ribosomal subunit rRNA binding	ribonucleoprotein complex biogenesis	intracellular non-membrane-bounded organelle, obsolete cytoplasmic part, protein-containing complex
SlPLATZ18	structural constituent of ribosome, protein binding, large ribosomal subunit rRNA binding, flavin adenine	ribonucleoprotein complex biogenesis	intracellular non-membrane-bounded organelle, protein-containing complex, obsolete

	dinucleotide binding		cytoplasmic part
SlPLATZ19	protein binding, selenium binding, serine-type endopeptidase activity,	carbohydrate utilization	virion component
SlPLATZ20	peptidase activity, transition metal ion binding, oxidoreductase activity	defense response to bacterium, obsolete oxidation-reduction process	virion component

Table S10. List of the PLATZ protein sequences used to construct the phylogenetic tree.

Name	Accession	Sequence
CrePLATZ1	Cre02.g095950	MEQQPRYEAVGSPEGAAGPLAAALMQPQSQQGYAQEFPRPSAMQQFEASHDGASASGGRH<br>EPAELAPSAAPVAFWKPLPVAAAGGGPAAESGAPEAAAFAFGAPQPPQIMPEAGHSMPVG<br>AGGAAAEAAEARQPGGSSADVAWGQQPQEQHPAAAVWHHPQVQPHTHLPPPQELPPQHLP<br>PPQPQQQPPTPQHLQQQLLLYPPQHQQQPAGTSAPAVLDAGGAAATAGGAAANGGDGCAM<br>WVGGGGTMRGDTDSVGPDGSGGASGAGAGAAAAAAAAAAAVGLAAASALPQPLHGYAFPPP<br>PFPAPSVQGLPPAPPSHEQQQQEQLLQQHHMQQQQMLQQHQLQQHQLQLQQQQMLQQHEH<br>MQQQFLQQQQQEQQEQQQQVVAMSGSPPHMLLSPFVPLYALGTAGGGGGGPTPAQAEAPL<br>GGLEQPNNPMEMQQHLTLQHWGSGAGAAAASALPPAGPGGGSFHGGIRSAQQMPCFPSAG<br>DLQNQQQQQLQHNAHQQQQQCQQQQYQQQQGQQQQHQIFHPQPHLAAGGASSLLVYPQTP<br>AAAASAAASADASAGAGAGGYLPPPALAPPDSQQLMPPQQQQQQQLLHEQHVRMLAAMAP<br>PPPAARGDAASPPLPMPSIFPPSGLTPPPPGSFHQLPPPLSSLGHPQQLQPQSQLQQLQP<br>SAAAAAGGFGVERSISLSGYLLLPQHSSQQQAKMQQQQQQQEQQQQAQMQQQQQMQVQQA<br>QVQMQMQQAQAAQMQMQHQQQQQAQQAVAAAASAAAAAAVDYFYSNMNHQQQQQLQQQQD<br>MLQLQDMQQQPYSYLQPPTAVEPAAQDTSGVSMPPPPPPPQIPQLPSLSLQPFQPPHLPPL<br>KATGRRLDAGGGSGSSAGGDQQPQQEGEGEEGIDTGTPTLVARRASPFALQSPPPMARWQ<br>AAHGGGGGGSDGGGNGAAPVAGVAAGVGASGTVIVAHAGGLEEQGHQHQQQLMQQQFTQA<br>YPQQQPSQQQIFVSYGGSGVLPPQFPSAGSLGDVNAAAAAPTTGTAAATHPMPQQFSGYV<br>PMAMPPPPQQQQQQQVSMAAAQPQPPPPLLPPAEANHYNPPPYNQQQLQQAPPAGMYDQH<br>TAHLMLYGMQQQLPIHAASVPLPAAEPRRAALLPPEQQAQALLQPAPSAPPVLISSDYST<br>GQALLQPSSVGGGGGGGGGAGGGDGGMFIGTVGRRVSLAGGGYVLSQHPDGGGGVGGSGG<br>VGGSGGAGGADAGSFAAGSGAGYLAPLAAAAVAQVAVGPYEYGQHQHVFQQQQQPQQQQP<br>QQVLSAFAPALHPNGGDAGSYLHQHQQEPRVGGGGDGTAEATPPPSVLLGGPGGSPPRPA<br>STAQAAMSFLMQVLDDGGDTRMTAEEVHALMHPQHTPPPPSLQQQQLQPQLQPQLDMGRT<br>ESLETTVATLGAAAAGTAPRGSAGTGAAVGLVAAPAGGGNEGASGPRGGSGSGGAGVGVG<br>AGDVPSPADGGGTDAGAQLQRLAVPRIALAVAERSGSSSIEGAAAAAAAAAYAHTRAATP<br>AVQSWTDAAAAAAAADGAVPTAAVLQRQQQQQQRQETEAPAVAEVAPQPSLLLPRAGLRP

		PSPPLSPISMQLEGAGSLLQPLLLPTPQRHHNHQHPVLLQPPQANAELGQAAAAAAGAEN<br>RREQQGVLGGVPAALSAGLTPAAAPLPAQATGSSGPGAEGSAAAAGEELQRQQLWKASWQ<br>TAGYDAVGRDVASHAASLVGSAATNVAAAAAGAGVAQPGALGAADAELGGFAAETKLEPS<br>PQQQPQLTLPAPPPPRPWSVLPSPAQQPQPQPQPQPGHAAGPTGLLPATPSAWPLPPLQV<br>RRQKQPPPGHSAAAVPPAAPAPAAAGAGGGAGASDAAGGGTASPPARKRQRRSAPQQPAG<br>AAATSAGPGGSSLLGTGPAAAAAAAAAAAAPPRSPAAARRRRQPREHGNPDLARDGVGGG<br>GGAGGGTSITAQRTPPPRAAGGGGRATNAPGGTSGGGAAAASGAISADDGDGAVDGDGFH<br>PQWLVVLTTDPRFFQPCPNPGCNPAHPGSRDALMTYLDVTDPYTPGVCSYCRPGRMAAGH<br>RLLQVRRSTYHEVVQLADVDELLDLEGVQLYTINHARVVFLRPRPQARAPKGAAMPSKCV<br>VDGRQLMDAGADYCSLRCKMERDPDHVFKDAACPAAAAVRAHIAARGAAGDAAGAGGAAG<br>AGPAGAAAATSAPLPAPSTAALLGFSPTAALAGARSPAAGGGAAAGAAASAAALPVVPLS<br>GRPTPAHARGGAPRGPFTYEDWVGGAGAAGAAAAAGAAVMSKAREVGGAAAGGGGGGGGG<br>VGEQAVVLEQEERALGRASGSGGRTGGDRGGQSPAAKRRRVPPVAPAALPPPLHPQQPLV<br>VGPEEAATAHGAGAGVMQLTPIRSPGPRASRQQQQQLLQLQHLRLQQQVAAAAAGSSGGV<br>SGSGDDIAAAAALRTPTPPPPRSEQSLHLLPDTHPPPPPRPGLLHVVPARSPAHSPRSHL<br>SGGQGNEVPQLFQAPSPQPPPLLRPHMPSPLRGTPPPSPKRSPSQPAAQPVHVTPYGTTG<br>GGTAAGVFGEDVRTSGGSGGGVVGAKTMSTTGDSHGGGGGAGMMAAAAAATPALSTGADS<br>LAGRGWEQGGGDGDSLGGGGDIVGQPDEQGAAATGLAARAQLAAASGATGAAVGGEEVGP<br>EVGPEGEGEREGEGEAVPPAHMLVGHTEEDRRATSLSASPPTTASGGAAAGTPPDTATAA<br>AATSTAAAATAAAAAAAAAALSSSGGVGGYGSDGVEGPRRRHHGIARGGPPPQLRPALLK<br>RSSPQQQQQQQRQVPPELAPLPLPPSPPSPATALRHAHFGPAPASAPGGVSALPVADDPA<br>VKSAAATLTARHAEPGQPLTPVEGTPPPSPLAAVPPASSYTGGGGDGGITGAVAALLDSG<br>RQESVNLGGGGGAAGGDSGWREESGSAPTEEDADGAHTSLTATATPTASQGNAADADAMD<br>ADAAAPSQDGLIVMAALPPLTAAAAAAPPPGQAPSPYASPRSATGAGGGRVLRHGSLSSG<br>SVGSWAAGMGPGGGGGGGGGATGSRGLPPLPGLEADFSTGWGALMRPHLAGMMATPAASE<br>AADGAQLAAADTAAAATGPAAEAAPQQQQQQAGVSGGAPPSLVPSPARSQPLLQVPLPLL<br>PPPHPELLQPQPAHRSAAATPPPAGLPPLQLPPRSPGRSPALSPGRSPARSPGRGNGGGG<br>GNGGGGSRTSRGSYSHAHSGLSHMDSGDTAGGAGGYSPGLGQGQGPHTPPGAGGGGGAGG<br>GGGGGGGGGGGGGSGPPSPHQQLLLMQLHPHHHKRKGAPHRSALE
CrePLATZ2	Cre12.g502251	MYIRRPQTANLKRASEESISTTALAGDAGADSGGGAGGGGGRVTAGAVVPPALVTLKSSS<br>SRQRSSSRLRVNGSSSSSSSRWIDPSGGNGAGSSSAAGSRSCTPRRQRPPDQAATTRTAT<br>AAAHAPSQPPPPRLPQHHDQQRPQLTSEATGLPLLTPQQLRDFLDATRLLLEPGARSSSG<br>GGGTSLTAVAFPAGGAAALSRSSSAGSHGRADSDCGGMEEGAGRSSHDGGGGGAAEAAAG<br>SNSCRADDAAGHLREAFSDPAEPGDGGAGSGGGGGGGVGDPGPDGPAAPAQARHSCYDEV<br>IRAADAAGLYDISGVQHYTDHGHKVVFLHARPQKSKPGAVSECGHCHRSLMDAGSRHCSL<br>ECKLNWQQRAPPLTQEQAAAAARATYGRRRARLVRVTRCRGRPDLPLAQLARTASPAAAR<br>PTATSATAAAAAAAAGPQLGAPDVDMQDAAGAGSVEPGRQQEAGAADGAAFASLLAAAQQ<br>ECRPQQGQQGSPPQRLATGQDDVVMMHVGQEAAGAAPAVAHAGCSTGSSAGGGTGGGTGG<br>GTGGGSAGAVAGRTDLVGHLPSSLPPRPPRKPLTGTALAGRPPRPSPLARQSLATAASTG<br>ETRAGPGCSMAAAPAAAACALATASQDPNPAVVQAPAPAAAPHQATSGTAGSDGVSDGGD

		GHAAAGHITGNSSSVGTCGGGGGGAGSSTAARATLLSRALAVALSGAEDKRAMCQAVAAA<br>VAATAPRSATLQALTAGTAEPQSGPPQRLRSQGSAGLAPAGASSSSSSRDGSGEGRRLSG<br>GRSDGGGSSGSGSGSGDCQVQERGVGAAAVAAAAATCAPALVGGLPPPLPAPGAVPSHAV<br>KAEAPLGPIGAVGPVGPVNSDAAVAAVTAACAGGGPAASRQLNDPQHQHHHQHQHQRHQQ<br>RQGDSAMEDVACRQPVPHAAPAAGSDRDGAAAGPASSGATGANDGAVRTAATSGPQAAAP<br>AAAVPPPSLPAARPATNNAAAASSPFGCFGGQAATAAAVAAVPLISLNPARPIASKSRAT<br>VKTGVPGAAAAAATAAAAQLPPHWLRPPLPPTARAAPGARGTAHCAGGGKAASLGAPPAP<br>LPQSSRRASPTATHDAGASGGGDMHGAGGVRAAAAAVHVQHVRHTGVKRRCVSMEPGGVL<br>PGGGGGYGGSGASELPEVTSPERRTGQRVCSLPNAQRAGGASPAAAAAAEADALAAHMAM<br>LSPSRLQRPLLPWERRAPAASAGGSGETADMETAPAANVAVACSRRHSSSAVWPDADIPG<br>LSGSNNFIGRVGAEGTGDTCSAQCVAGGCGNLARSSSGAGGGGSGGGGGGGGRGRRSSSA<br>TARARPRRGSSSSAKSPPVPASSAAVAPATHVQAHPAATAAATPAVSGAEGAVADAAAAA<br>AAAVTANSSRRVKARKGRPHQAALV
CrePLATZ3	Cre16.g650400	MLAACPPLGAVASPFKGPSAATNYAYDADVSDERCSSDETSDAFDTNVRCALRPGAQATG<br>ATGKTMNQRPVAVCASWVPSFLSATYFQPCERHRHHKKNECTFFCISCGAKPHSVCQHCM<br>GAHAGHQVIQVRRYVYCDVVRACDINAYVDTTGVQNYIINSAKVMFLNHRPHSKIGRVNG<br>ADTCRTCHRHLREGFSYCSLACKVEALHLTCGGAAISSGAAGSASASAGGDAAAAQPPPA<br>AAAVGPGAVSLIRAGSDITGSAAAAVAAAVARQQPVSPATATAAAAASGVSPFALEPAVA<br>AAAGGAVAAAASCAALSSDPESSEPEAVEEPFMARARSLGLSAAAAAGVAAATAATSALS<br>PLLPQRRRVGAPAYVLSGRRYMAAAAAAAAAAAAAAAAAAAAAAAAAGGGGSSSSASDCD<br>VDDMGMEEEEQQQQQQQQQLDANFENPPKRRRSAVISGRRGVGSSSSYGVMAAGGPTALA<br>AVTLLMPHHSLGLTLPPPPPSLAAAPSRATPPPPSRATPPPPSAPKPQPVAATGGNGIGH<br>MSRNSSMPSLPAAATAAGGAGSAGAAAAAAAAAAAAAASSNSRRKRSMPQRAPSL
PpPLATZ1	Pp3c10_12560	MAELEQSSCGSLREGPVWLSPLLQTEFFGHCKKHTTGKHEKNQFCLSCCSGPYCPQGLSQ<br>SHSGHMSIQVRKASHRDVVRITDMQKYLDLSNIQPYTINGAKIVFLQSRPQPKLCKGAAT<br>YCDTCHRSLADQVRFCSINCKLVAIQDASEQDTELKLSPTTEFQSVQGGARKGGSCGYLR<br>LSITDGTDSANHETTSFSPSTPKRQGSEQETSLSGLEMPPFKRQKTLEDVLCPFSPMSVL<br>GSGAAFGHDWIDCYGPSTPERSSELSPRMHSRKQCHPVRAPFF
PpPLATZ2	Pp3c13_4760	MGEGDINLSEGPLWLKPLLKADFFATCAVHGVSAKSECNLFCFNCMGDGICASCTADHKD<br>HHVVQIRRSSYHDVIRVSEIQKLLDISTVQTYIINSARVVFLNERPQPRTAKGVTNTCET<br>CERSLLDTFRFCSLGCKLAGIKKHKELSFLLQPKSQGFGSTNSRVPSDSEDSSTHKKATR<br>SRKVLQLQSSPLSHTSFESSETDHEIVEDPTFAKRRAWHQGEGGQSSLGVTSGCFHDTVG<br>SMPVQLVVDMSPRTPPRSNVLRIAKRRKGIPHRAPFRA
PpPLATZ3	Pp3c15_23190	MVMGGTFMNREGPSWLPALVKCDDFFSHCNHHTSGKNERNQFCFDCPQEGPLCPEELTVS<br>HRGLGHATIQIRRASHRDVVRIADIQKYVDLTNIQPYTINSAKIVFLQSKPQPKIVKGAA<br>HYCERCQRSIADPVRFCSISCKLEGIQLDPHDFTLTLTVFSKSGPGFFSKGGTDNVGAAS<br>PEHSVHSHGSGVTDFSPETPKKISKRSLKETPSPFSKKAKSALSAAPVFGLLVHLPRDDG<br>MGLTLTSPMAPPTPTYESGPKVHHRKQVRPHRAPMF

PpPLATZ4	Pp3c18_12180	MVGYDLSVLPQKPAWLESLLAEKFFVSCAKHGALKKNERNVFCVDCNAGVCQHCVPAHQN<br>HCILQIRRYVYHDVIRLQDIQRLLDCATVQTYIINSARVVFLNQRPQPRPSKGLGNACGT<br>CDRSLQDSYAYCSVACKVDAVISNGKDLSSLLPESGNMPYSFFTCSPTRSLKGGKHELED<br>EVLTDSPFDMSPTHTSSASTGSEGAGFCGVASTASTQLLPKKVRSGRISAIATSPKSVIF<br>PVSVKRRKGTPHRSPFC
PpPLATZ5	Pp3c18_12270	MVGYDLSALPQKPAWLESLLAERFFVPCAKHGAFKKNERNVFCVDCNAGVCQHCVPDHQN<br>HCILQIRRYVYHDVIRLQDMQRLLDCSTVQTYIINSARVVFLNQRPQPRPSKGLGNACGT<br>CDRSLQDSYAYCSVACKVDAVVSSGKDLSTLLPESGNMPYSFFTCSPTRSLKGGKHELED<br>ELLTDSLCDGSPTHTSSASTGSEGIGFCGIVSTASTQLLPKKVRSGRISSMATSPKSVIF<br>PVSVKRRKGTPHRSPFC
PpPLATZ6	Pp3c21_11530	MVGYDLSAIPQKPAWLESLLAEKFFVPCAKHGALKKNERNVFCMDCNAGVCQHCVPAHQN<br>HCILQIRRYVYHDVIRLQDIQRLLDCSTVQTYIINSARVVFLNQRPQPRPSKGMGNACGT<br>CDRSLQDSYAYCSVACKVDAIVNSGSDLSSLLPVSCNMPYAFFTCSPTRSLKGGKHELED<br>ELLTDSPSDESPTQTSSASTSSEVTGFCGVVSTASTLLLPKKVRSGRLSAMTTSPVSVIF<br>PVSVKRRKGTPHRSPFC
PpPLATZ7	Pp3c21_8120	MVGYDLSTLPQKPAWLESLLGEKFFVPCAKHGALKKNERNVFCVDCNAGVCQHCVPAHQS<br>HCILQIRRYVYHDVIRLQDIQRLLDCSSVQTYIINSARVVFLNQRPQPRPSKGMGNACGT<br>CDRSLQDLYAYCSVACKVDAVISSGGELSSLLPGDGSKPYSFFTCSPSRTSKHELEGELL<br>TDSPFDVSPTHTSSASTGSEGAGFGGVVSTASTLLLPKKVRSGRVSVMATSPKSVIFPVS<br>VRRRKGTPHRSPFC
PpPLATZ8	Pp3c26_11480	MGGPVVAEEDTTLVEGPVWLKPLLKADFFATCPLHGVSAKSERNLFCFKCMGDGICASCA<br>VDHKDHHVVQIRRSSYHDVIRVSEIQKLLDLSTVQTYIINSARVVFLNERPQPRPAKGVT<br>NTCETCERSLLDTFRFCSLGCKLAGIKRHKDLSFSLQPKPQGTVSTNSRVLSDSEDSSTQ<br>KKAPRSRKVSQLRSSPLSHTSFEGSETEHETTEDTTFNKRRVWLRIDAGPSSPGASSERF<br>NRATSSLPVLVVDMSPRTPPRSNIHRIAKRRKGIPHRAPLRA
PpPLATZ9	Pp3c3_19680	MAHLEQSTCGLLQAGPTWLSPLLETEFFGYLRKASHRDVVRVADIKKYLDISNIQLYPIN<br>GAKIVFLQSRPQTKSCKGAPKYCDTCHRSLADQVRFCSINCKRVAVCDASVRDLELTLSH<br>SIDSVSDGTDSMNLEATNFSPSTPNRHGNHTELNNLSLKMPPFKRQKISEDRSHSLSPMS<br>VLGAGVAFRFDCLRFTAPPRPSDTVNLVQECTAGSSSTRCGRHSFELFNDIPLKAEHCEN<br>ASLIVHYFRQWIYVHALQLQSK
PpPLATZ10	Pp3c3_3180	MVSPSPSRTAELHAVQGGPVMAEEDTNLAEGPLWLKPLLKADFFATCGIHGVSTKSECNL<br>FCFNCMGDGICASCAVDHKDHHVVQIRRSSYHDVIRVSEIQKLLDISTVQTYIINSARVV<br>FLNERPQPRPAKGVTNTCETCERSLLDTFRFCSLGCKLAGIKRHKELSFFLQPKAQGVVS<br>TNVRGPSDSEDSTHKKAPRSRKVSQLQSSPLSHTGFESSDTDHETMEDTTFKGRTLHHEK<br>VGQSSPGVTSGRFGDKNGSAPVQLVVDMSPRTPPRSNVLRVAKRRKGIPHRAPLWA
PpPLATZ11	Pp3c4_12320	MESVAQLEHSQSNGNSQRQGPAWLSPLLRKEFFGHCKKHTTGKHEKNQFCLTCCSGPYCP<br>EGLSQSHSGHASVQVRKASHRDVVRITDIHKYLDISNIQAYTINSAKIVFLQSRPQPKLC<br>KGAPKYCDTCHRSLADQVRFCSINCKLVSISHELTGGSATEETLSPPTDARSISGNPTQS<br>GEGSSHLAPSVSNGHDGESAMNCETATSFFPVTPKKQGTKRRSSPSCKLRLVVPSNKRKA

		LEDGAAPLSPMSVLGSGPAFAQDWPDFHGLSTPDSLDDSSTRIHPRKQFHPSRAPFF
PpPLATZ12	Pp3c4_30530	MGGQVMEEGDVSLVEGPVWLKPLLKADFFATCALHGVSAKSERNLFCFKCMGDGICASCA<br>VDHKDHHVVQIRRSSYHDVIRVSEIQKLLDISTVQTYIINSARVVFLNERPQPRPAKGVT<br>NTCETCERSLLDTFRFCSLGCKLAGIKRHKELSFILQPKPQGWFSPSSRVLSDSEDSSTH<br>KKAPRSRKVSQLQTSLLSHTSIEGSEETDHETAEDAACNKRRPWQRVEAGLLPSPACSAE<br>HLHRATSSLPVLVVDMSPRTPPRANVLRIAKRRKGIPHRAPLRA
PpPLATZ13	Pp3c8_4000	MWVHLVVNADHRIWFRRWDEAEHSPRSTTTTPVLGELWIRSKGRTNEMGPGWLKPLLKTS<br>FFASCRIHGLSSHKGECNLFCLQCMGDSMCSLCLPSHKDHHVVQIRRSSYHDVLRVSEIQ<br>KVLDITCVQTYIINSARVVFLNKRPQPRPAKGVTSICEGCGRSLLESYRFCSLGCKLGGM<br>ERNLELTFMPRHQPLMSIDNHMHPSSTLNSEESSFHRKAPRTKKLSLLPAPSSPVCTVCD<br>GQEIRDEASTVSQQCDSHLNSKTHNCVINVQSCRRPACLTATVYTRSAKRRKGIPQRAPL<br>GP
PpPLATZ14	Pp3c9_19170	MVMGGMFMNREGPSWLPALVNSENFFSQCNHHTSGKNERNQFCFDCPQEGPLCPEGLAAS<br>HRGQGHATIQIRRASHRDVVRISDIQKYVDLANIQPYTINSAKIVFLQSKPQTKIVKGAA<br>HYCEWCHRSIADPVRFCSISCKLAVIQQDPHDFTLTLTMFSKSGPGFFTKGCTVSVGAMS<br>SEHSAHSHGSGIMDFTPDTPMKNSKRSAKDTQWPLSKKVKVALSASPSVGMSVLLPRDDE<br>LRLSSMSPMAPPTPTYESGPKVHHRKQVRPHRAPMF
AmtrPLATZ1	AmTr_v1.0_scaffold00002.219	MAIEQELSLKEIRPKNRRIMGGRSPDEENIWPPWLKPLLETSFFVQCKFHTDSHKSECNM<br>YCLDCSNGALCSFCLQHHKDHHVIQIRRSSYHDVIRVSEIQKVLDISGVQTYIINSARVV<br>FLNERPQPRPGKGVTNTCIICHRSLLDSFTYCSLGCKIAGTSRKHGRVNMAKEATVSDTE<br>ESYTNVQMSFSPPTPPPTVFSYRSVKRRKGIPHRAPLGGICLDY
AmtrPLATZ2	AmTr_v1.0_scaffold00008.128	MEVQTVMARPPWLKPLLGTSFFRQCKSHGDSNKNECNMFCLDCISDALCSSCLLVHRHHR<br>IIQIRRSSYHDVIRVSEIQKVLDIGGVQTYVINGAKVVFLNHRPQPRPGKGVTYACEICD<br>RSLQDPARFCSLGCKMAGMTSNRKSSEDTHIASSQCMAPQTPPSSCRTRRRRKGIPQRSP<br>MGSC
AmtrPLATZ3	AmTr_v1.0_scaffold00022.21	MVSVFSLGLSQRSARLNLSYVTRLGFGICSYCVSLLTGKLAIENQTRFGLVGCKRKNVMV<br>GPMIRPGNEELYPRWLKPLLQTSFFIPCLVHHDAHKNECNLYCLNCMGNALCSYCLAHHK<br>DHHVVQIRRSSYHNVIRVSEVQKVLDISAVQTYIINSARIVFLNERPQIRAGKGVTNTCE<br>ICNRSLLDTYRFCSLGCKLGGIKRDPELTFALRPKQQREAAQGSESEESSTPKRSTKRNT<br>FQRFMDENPDYMMRYGEKYSSSSDEANNFTSFISPATPPIINYRNSRRKGIPHRAAPF
AmtrPLATZ4	AmTr_v1.0_scaffold00032.279	MVREEEEEIEDEVLAPPPWLDGLVKGSFFTPCAFHESRKKNEKNVFCLECCVSLCTHCIS<br>SHPPHPFLQVRRYVYHDVVRLDDLEKLIDCSRVQPYTINNAKVIFLNRRPQTRASKGSNN<br>ICLSCDRMLQDPYSFCSLSCKVDFLVHRGEDLSSVLYKYRDIHFAFAQFEGLRMDGPEDH<br>SNPSSVLEDPAMAGNHSGSAEEDGGVVDQNTASSAETPDLSKKRGFLKRSFAPFLRRRKG<br>HPHRAPFS
AmtrPLATZ5	AmTr_v1.0_scaffold00033.111	MVFCRGFRSLHAHESAKKNEKNIFCLDCCTSICPHCLPAHRAHRLVQVRRYVYHDVVRLE<br>DLEKLIDCSNVQAYTINSAKVVFIKKRPQNRQFKGSGNVCTNCDRTLQEPYIHCSLGCKV<br>DFVLKHNKDLTAYLRTCKTLQLSPDFLVPHDAMEEEMSETPHSVMDCDEAMGLSSGSSGC<br>DNNLAFACTATTDLVRKKRTGSYYVCARSANVENEDMVVTMSRRKGIPHRSPLC
AmtrPLATZ6	AmTr_v1.0_scaffold00059.90	MPDHAQYPPWLDTLLSEKFFSPCLLHESSKKNEKNIFCLDCCTSICPHCLHYHRSHRLLQ<br>VRRYVYHDVIRLDDLEKLIDCGFVQSYTTNSAKVVFLNQRPQSRPFKGSGSVCHTCDRSL

		QDPYHFCSLSCKVSYLLRYEGCVSRYLYECEYLPLSDFVKEMEDGQMTPISVLDGGISLR<br>TSSGSSANGAVECRTLACTATTETVTRRKRTAAISSRNRQIAAAIRATCQVVSRRKGVPH<br>RSIGN
AmtrPLATZ7	AmTr_v1.0_scaffold00138.15	MRFICGRWFVEERTDEISLVSRSVILVFSSSSAAISKARAKVRRYVYNDVIRLQDMHKYL<br>DCYQIQTYRINGAKVVFLNARPQTKLTKVLPSKCRMCDRGLKDGYKYCSVSCKVKDVDAN<br>KGSNALCKWLPLPEFISFPGKDDGSEGKEDVSGISSCSQDEEEPEEEEELTGSIEEEAFD<br>ANPQWLLPVLKPKRKLHKRKGIPRRSPVY
SlPLATZ1	Solyc01g091000	MLDYAQNLPQWLITLLTEKFFNACIIHEDAKKNEKNVFCLDCCEGICPHCLSPHRSHRLL<br>QIRRYVYHDVIRLGDANKLLDCAFVQSYTTNSAKVVFLNQRPQTRASRGSGNCCIICDRG<br>LQDPFLFCSVSCKVQHILKTEGKLTKYIYRCEYMTLPEPGLDDGQMTPDTILEPIGSVRT<br>ESGSSGSGGAAEEVGCRTVGCTATTEVVRKKRSTLSAFRSVFQPGCGPGSGISVSMLNRR<br>KGTPQRSPLY
SlPLATZ2	Solyc02g033100	MNSSNSSHVETNEQGELEPSRNMPTWLGPLLKKTFFGACLVHDELEKNELSKYCITCDSY<br>LCKHCICTNKHNDHDKLKIYRHVYKDVVLLEKMEKYIDCKLIQPYKCNKKLVIALNPLPH<br>CGSGSLIVGDPTCLTCKRRLHDPKLFQFCSIACQVEAKWGKIVETKRKRKRKGIPHRAPL<br>K
SlPLATZ3	Solyc02g033110	MSSSDSSPFETNEQGELKPSRSMPTWLGPLLKKSFFGECLVHDGLQKNERTKYCITCDSD<br>LCRYCISINKHNDHDQLKIYRHVYKDLVPLEQMKKYTDCKLIQPYKCNKKWVIALNPLPH<br>CGSDSLIAGDPTCLTCKRRLHDPKRFQFCSIACQNSGNYA
SlPLATZ4	Solyc02g033120	MSSSDSSPFETNEQGELKPSTSMPSWLGPLLKKSFFGECLVHDGLQKNERSKYCITCDSD<br>LCRYCISTNKHNDHDQLKIYRHVSKDLVPLKQMKKHIDCKLIQPYKCNKKWVIALNPLPH<br>CGSGSLIAGDPTCVTCKRRLHDPKRFQFCSIACQ
SlPLATZ5	Solyc02g036120	MARTFEEVIHDGLQKNERSKYCTTYDSDLCRYCISTNKHNDHDQLKIYRHVYKDVVPLEQ<br>MKKYIDCKLIQPYKCNKKWVISLNPLPHCGSGSLNAGDPTCLTDPEWFQFCSIACQVLNY<br>LSLRFKFSGCLGMIFILLFDIFFEIEFWKLCWIKNSPFTFTR
SlPLATZ6	Solyc02g036130	MGSSDSSPFETNEQGELKPSRIMLTWLGPLLKKTFFGECLVHDRLQKNERSKYCITCDSD<br>LCRYCISTNKHNDHDQLKIYRHVYKDIVPLEQMKMYIDCKLIQTYKCNKKWIIALNPLPH<br>SWSGSFIACQVLISPSV
SlPLATZ7	Solyc02g036140	MGSTDSSPFETNEQGELKPSRSMPTWLGPLLKKTFFGECLVHDGLQKNQRSKYCITCDSD<br>LCRYCIATNKHNDHDQLKIYRHVYKDVVPVEQMKKYIDCKLIQTYKCNKKWIIALNPLPH<br>SGSGSLIVGDPTCLTCKRRLHDPERFQFCSIACQVLIPPSISNLVVICQ
SlPLATZ8	Solyc02g036170	MSSSASSPFETNEKGELKPGRSMPTWLGPLLKKTFFGECLVHDGLQKNERSKYCITCDSD<br>LCRYCISTNKHNDHDQLKIYRHVYKDVVPLEQMKKYIDCKLIQTYKCNKKWIIALNPLPH<br>SGSGSLIAGDPTCLTCKRRLHDPERFQFCSIACQVLFSPSVSNLVVVWR
SlPLATZ9	Solyc02g036200	MSSSASSPFETNEQPMSPLSSFVRGELKPGRSMPTWLGPLLKKTFFGECLVHDGLQKNER<br>SKYCITCDSDLCRYCISTNKHNDHDQLKIYRHVYKDVVPLEQMMKYIDCKLIQTYKCNKK<br>WIIALNPLPHSGSGSLIAGDPTCLTCKRRLHDLERFQFCSIACQVHFSPSVSNLVVVWR
SlPLATZ10	Solyc02g036230	MSSSASSPFETNEQRELKPGRSMPTWLGPLLKKTFFGECLVHDGLQKNERSKYCITCDSD<br>LCKYCISTKKHNDHDQLKIYRHVYKDVVSLEQMKKYIDSKLIQTYKCNKKCIIALNPLPH<br>SGSGSLIAGDPTCLTCKRRLHDPERFQFCRIACPVEEKWGKIAETKRKRKRKGIPCRAPL

		K
SlPLATZ11	Solyc02g068510	MEEMMKPAWLQGLMSENFFGGCEIHQNRRKNEKNIFCLECCQSFCPHCLPQHHSHPLLQV<br>RRYVYQDVIRLDDLEKLIDCSYIQPYTINSAKVIFLNQRAQSRSCKASGNSCFTCDRVLQ<br>NPFNFCSLSCKVDFMVYQGEDLSNIICKFDDSEFALSQFEGLHVDTSDLIDEESQITPNS<br>ILEDPLECRGSSCSNNVRGNSGISHDGRMVKNKKKSSGFFPGLVLSLSNRRKGAPQRSPL<br>S
SlPLATZ12	Solyc04g008090	MLIGGEIRVRSKVDWIENLLNIKFFESCGYHRELRRNEKNMFCIDCNLCFCKHCVSSSSH<br>CFHEWLQICKYVYHDVVRLHEIQKHLNCAEIQTYKINGEKAIHLNPRPQSKDSKTSKLKG<br>SVTCEACGRHLQDLPNRFCSIACKVSVDANICKGRQKNYISNQITKFDHSNENESCISLN<br>ESSEVIQTWCISPLKPKKNLHKRKGVPRRAPIR
SlPLATZ13	Solyc06g061240	MGPDEDDNRWPPWLKPLLKERFFVQCKLHADSHKSECNMYCLQCNNAPLCSVCLAHHRDH<br>PVIQIRRSSYHDVIRVNEIQKYLDISSVQTYIINSAKVVFLNERPQPRPGKGVTNTCEVC<br>ERSLLDSFKFCSLGCKIVGTSSNFVKKKKKPKNSPEKKRLPAAVVAASEFDDSYSSSSSS<br>HGRRNKIQSFTPSTPPPTSANYKTAKRRKGIPHRAPTGGLFIEY
SlPLATZ14	Solyc07g007320	MGIQKPTWLEALYREKFFAPCSIHESAKKNEKNICCLDCCTSICPHCVMAHRFHRLLQIR<br>RYVYHDVVRLEDLEKLIDCSNVQAYTINSAKVIFIKKRPQNRQFKGSGNYCTSCDRSLQE<br>PFIHCSLGCKVDFVLHHYNDISPFLRRCTTLQLGPDFFIPHDMADYDTANETAQSTIVDN<br>DEPWGSSLTSGSSSGSENMMLMSTTNNFPCVRKKRSGLYVCGRITINSYNKNISDEDMAT<br>SMSRRKGIPHRSPLC
SlPLATZ15	Solyc07g049120	MPAWLGPFLKKIFFGTCLVHDELQKNELNKYCITCDSDLCRNCVATDEHNEHDLLQIYRH<br>VYKDVVPLDEMEKYIDCTKIQPYKCNKKWVVALNPLPHNGSGSLIVGDPTCYTCKRRLND<br>PEQFRFCCIACQVEAKREKPVETKRKRKRKGIPHRAPLK
SlPLATZ16	Solyc07g049130	MPAWLRPFLKKTFFGTCLVHDELQKNELNKYCITCDSDLCKNCIATDKHNGHDLLQIYRH<br>VYKDVVPLDEMEKYIDCTRIQPYKCNKKWVVALNPLPHSGSGSLIVGDPTCYTCKRRLND<br>PEQFRFCCIACQVEAKREKPVETKRKRKRKGIPHRAPLK
SlPLATZ17	Solyc08g005100	MGVGGADEEENNWPPWLKSLLKERFFFQCKLHIDSHKNECNMYCLDCINGPLCSLCLSHH<br>KDHIVIQIRRSSYHDVIRVNEIQKYLDISLVQTYIINSAKIVFLNERPQPRQGKGVTNTC<br>QVCERTLLDSFKFCSLGCKVIGTSKNFIKKPRDLSAKRQLKAIASDSDDPYSPNNHSRYR<br>STIINKVQSFIPSTPPPSSVNYRTAKRRKGIPHRSPMGGLVIEY
SlPLATZ18	Solyc08g076860	MGAGGPDEEDNRWPPWLKPLLKERFFVQCKLHVDSHKSECNMYCLDCMDGALCSICLSHH<br>KDHRAIQIRRSSYHDVIRVNEIQKFLDITSVQTYIINSAKVVFLNERPQPRPGKGVTNTC<br>EVCERSLLDSFRFCSLGCKIVGTSKNFVKRPKQSPEKKKSSPAAAAAVYDSEDSYSSSSH<br>GRQNSTSNKVHQSFSPSTPPPTSVNYRTAKRRKGIPHRAPMGGLVIGY
SlPLATZ19	Solyc10g085800	MTMLVPPWLEPLLNTAFFSICRTHGDAARSECNMYCLDCNDNAFCFYCRSSKHKDHQVIQ<br>IRRSSYHDVVRVSEIQKVLDISGVQTYVINSARVLFLNERPQPKSSGKASSHVCEICGRS<br>LLDTFRFCSLGCKLVGIKRNGDSSFILDAKNEVLALQRGEGISSRGGNQLREGLEHDNIY<br>PPTPPPPPSNARRRKGIPHRAPLGS
SlPLATZ20	Solyc12g010470	MGIQKPSWLKALYVEKFFVPCSIHESAKKNEKNVCCLDCNISICPHCVTSHRVHRLLQIR<br>RYVYHEVVRLEDIENLIDCSNIQAYTINNAKVVFIKKRPQNRQFKGGNYCTSCDRSLQEP<br>FIHCSLGCKVDFVLKHHKDLSPFLRRCTILQLSSDFFIPQDMGDDDMAIDTAHSTIVDND<br>EPWSSSSSTGSGSENMSFPCTEFVRKKRSGMHVCGRSANNCNNITEEDMATSISRRKGIP

		QRSPLC
StPLATZ1	Soltu.DM.01G031640	MNMLDYAQNLPQWLVALLTEKFFNACIIHEDAKKNEKNVFCLDCCEGICPHCLSPHRSHR<br>LLQIRRYVYHDVIRLGDANKLLDCAFVQSYTTNSAKVVFLNQRPQTRASRGSCNCCIICD<br>RGLQDPFLFCSVSCKVQHILKTEGKLTKYIYRCEYMTLPEPGLDDGQMTPDTILEPIGSV<br>RTESGSSGAAEEVGCRTVGCTATTEIVRKKRSTLSAFRSVFQPGCGPGSGISVSMMNRRK<br>GTPQRSPLY
StPLATZ2	Soltu.DM.02G004340	MPTWLGPLLKKTFFGTCSVHGELQKSELNKYCITCDSDLCRYCISTNKHNDHDQLKIYRH<br>VYKDVVPLQQMEKYIDCKLIQPYKCNKKWVIALNPLPHCGSGSLIAGDPTCLTCKRRLHD<br>PEQFQFCSIACQVEAKWGKIAEMKRKRKRKGIPHRAPLK
StPLATZ3	Soltu.DM.02G004360	MEEDKMNSSDSSPLETNEQGELEPSRNMPTWLGLLLKKTFFGACLVHDELQKNELSKYCI<br>TCDSDLCMYCISSNKHNDHDQLKIYRHVYKDVVLLEQMEKYIDCKLIQPYRCNKKWVIAL<br>NPLPHCGSGSLIAGDPTCLTCKRRLHDPEQFQFCSIACQVEAKWGKIAEMKRKRKRKGIP<br>HRAPLK
StPLATZ4	Soltu.DM.02G004380	MEEEKMNSSDSSPLETNEQGELEPSRNMPTWLGLLLKKTFFGACLVHDELQKNELSKYCI<br>TCDSDLCMYCISSNKHNDHDQLKIYRHVYKDVVLLEQMEKYIDCKLIQPYRCNKKWVIAL<br>NPLPHCGSGSLIAGDPTCLTCKRRLHDPEQFQFCSIACQVEAKWGKIAEMKRKRKRKGIP<br>RRAPLK
StPLATZ5	Soltu.DM.02G004390	MNSSDSSPFETNEQGELEPSRNMPTWLGPLLKKTFFGACLVHDELQKNERSKYCITCDSD<br>LCRYCISTNKHNDHDHVKIYRHVYKDVVPLEQMEKYIDCKLIQPYRCNKKWVIALNPLPH<br>CGSGSLIAGDPTCLTCKRRLQDPEQFQFCSIACQVEAKWGKIAEMKRKRKRKGIPHRAPL<br>K
StPLATZ6	Soltu.DM.02G011310	MEEMMKPAWLQGLMSENFFGGCEIHHNRRKNEKNIFCLECCQSFCPHCLPQHHSHPLLQV<br>RRYVYQNVIRLGDLEKLIDCSNIQPYTINSAKVIFLNQRAQSRSCKASGNSCFTCDRVLQ<br>HPFNFCSLSCKVDFMVYQGEDLSNIIYRFDDSEFALSQFEGLHVDTSDLIDEESQITPNS<br>ILEDPLECRGSSCSNNVMGNSGISHDGRMVKNKKKNSGFFPGLVLSLSNRRKGAPQRSPL<br>S
StPLATZ7	Soltu.DM.04G003470	MFCIDCNLCFCKHCVSSSSHCFHEWLQICKYVYHDVIRLHEIQKHLNCSEIQTYKINGEK<br>AIHLNPRPQSKDNKTSKLKGNVTCEACGRHLQDLPNRFCSIACKVSIDANICKERQKNYI<br>SNQITKFDHSNENESCISLNESSEVIQTWCISPLKPKKNLRKRKGVPRRAPIR
StPLATZ8	Soltu.DM.04G038210	MVSQDEIGPAWLKPLLKANYFHTCTIHGDSNKSECNMYCLDCMGSNALCSYCLTNHKHHR<br>LVQIRRSSYHNVVRVNEIQRYLDISCIQTYIINSAKIVFLNERPQPRPGKGVTNTCEICC<br>RSLLDTFRFCSLGCKLNGIRRGDEELTFTLKAETNEYWDDESSTPKKKQRRDEKVTFGFR<br>HLNIEAYNMSPDTYHNNSNRRKGIPHRAPF
StPLATZ9	Soltu.DM.06G018660	MGPDEEDNRWPPWLKPLLKEQFFVQCKLHADSHKSECNMYCLQCNNAPLCSVCLAHHRDH<br>PVIQIRRSSYHDVIRVNEIQKYLDISSVQTYIINSAKVVFLNERPQPRPGKGVTNTCEVC<br>ERSLLDSFKFCSLGCKIVGTSSNFVKKKKKPKNSPEKKRLPAPMAASESDDFYSSSSSSH<br>GRRNKIQSFTPSTPPPTSANYKTAKRRKGIPHRAPTGGLFIEY
StPLATZ10	Soltu.DM.07G002730	MGIQKPTWLEALYREKFFAPCSIHESAKKNEKNICCLDCCTSICPHCVMAHRFHRLIQIR<br>RYVYHDVVRLEDLEKLIDCSNVQAYTINSAKVIFIKKRPQNRQFKGSGNYCTSCDRSLQE<br>PFIHCSLGCKVDFVLNHYNDISPFLKRCTTLQLGPDFFIPHDMADYDTANETAQSTIVDN<br>DEPWGSSLTSGSSSGSENMMLMSTTNNFPCVRKKRSGLYVCGRITINSYNKNISDEDMAT

		SMSRRKGIPHRSPLC
StPLATZ11	Soltu.DM.07G016190	MPAWLGPFLKKTFFGTCLVHDELQKNELNKYCITCDSDLCRNCIATNKHNEHDLLKIYRHVYKDVVPLDEMEKYIDCTKIQPYKCNKKWVIALNPLPHCGSGSLIVGDPTCYTCKRRLNDPEQFRFCCIACQVEAKWGKIVEMKQKRKRKGIPRRAPLK
StPLATZ12	Soltu.DM.08G004010	MGARGPDEEENNWPPWLKPLLKERFFVQCKLHIDSHKSECNMYCLDCINGPLCSLCLSHHKDHIAIQIRRSSYHDVIRVNEIQKYLDISLVQTYIINSAKIVFLNERPQPRPGKGVTNTCQVCERTLLDSFNFCSLGCKVIGTSKNFIKKPRDLLAKRRLKAIASDSDDTYSHNNHGRYRSTTINKVQSFIPSTPPPTSVNYRTAKRRKGIPHRSPMGGLVIEY
StPLATZ13	Soltu.DM.08G022680	MGAGGPDEEDNRWPPWLKPLLKERFFVQCKLHADSHKSECNMYCLDCMDGALCSICLSHHKDHRAIQIRRSSYHDVIRVNEIQKFLDITSVQTYIINSAKVVFLNERPQPRPGKGVTNTCEVCERSLLDSFRFCSLGCKIVGTSKNFVKRPKQSPEKKRSSPAAAALYDSEDSYSSSSHGRQKSPSNKVHQSFSPSTPPPTSVNYRTAKRRKGIPHRAPMGGLVIGY
StPLATZ14	Soltu.DM.10G021510	MTMLVPPWLEPLLNTAFFSICRTHGDAARSECNMYCLDCNDNAFCFYCRSSKHKDHQVIQIRRSSYHDVVRVSEIQKVLDISGVQTYVINSARVLFLNERPQPKSSGKASSHVCEICGRSLLDTFRFCSLGCKLVGIKRNGDSSFILDAKNEVLALQRGEGISSRGGNQLREGLEHDNIYPPTPPPPPSNARRRKGIPHRAPLGS
StPLATZ15	Soltu.DM.12G027850	MGIQKPSWLKALYVEKFFVPCSIHESAKKNEKNVCCLDCNISICPHCVTSHRVHRLLQIRRYVYHEVVRLEDIENLIDCSNIQAYTINNAKVVFIKKRPQNRQFKGGNYCTSCDRSLQEPFIHCSLGCKVEFVLKHHKDLSPFLRRCTILQLSSDFFIPQDMGDDDMAIDTAHSTIVDNDEPWSSSSSTGSGSENMSFPCTEFVRKKRSGMHVCGRSANNCNNITEEDMATSISRRKGIPQRSPLC
AtPLATZ1	AT1G21000	MGPMIRTEEEEDYTSPPWLMPMLRGSYFVPCSIHVDSNKNECNLFCLDCAGNAFCSYCLVKHKDHRVVQIRRSSYHNVVRVNEIQKFIDIACVQTYIINSAKIVFLNERPQPRIGKGVTNTCEICCRSLLDSFRFCSLGCKLGGMRRGDLSLTFSLKGKHGREYLGGSESDEATTPTKMRKTNAFNRLMSGLSISTVRFDDYGPNGDQRSSSSGDEGGFSFSPGTPPIYNHRNSSRRKGVPHRAPF
AtPLATZ2	AT1G31040	MVREGEEEEEMMMMMATKPAWLEGLMAETFFSSCGIHETRRKSEKNVFCLLCCLSVCPHCLPSHRSHPLLQVRRYVYHDVVRLSDLEKLIDCSYVQPYTINGAKVIFLNQRQQSRAKVSSNVCFTCDRILQEPFHFCSLSCKVDYLSYQGDDLSSILYRIDESDFTFEGLRMDGHDQLGEISTMEDGEDILVISDESEQGNNSHKKEKKKSKKKKPESNYLPGMVLSSLGNRRKGAPHRAPFS
AtPLATZ3	AT1G32700	MGAEEETNKTYPHWLKPLLREKFFVQCKLHADSHKSECNMYCLDCTNGPLCSLCLSFHKDHHAIQIRRSSYHDVIRVSEIQKFLDITGVQTYVINSAKVVFLNERPQPRPGKGVINTCEVCYRSLVDSFRFCSLGCKISGISKKKRKEWTNNLSDSDDSYSSTSIGRLKKNDDIMNNSFTPSTPPLSAVNRRIAKRRKGIPHRAPFGGLIIEY
AtPLATZ4	AT1G43000	MENDDVMTPPWLTPMLRADYFVTCSIHSQSSKSECNLFCLDCSGNAFCSSCLAHHRTHRVIQIRRSSYHNVVRVSEIQKHIDISCIQTYVINSAKIFFLNARPQCRTGKSLNKTCQICSRNLLDSFLFCSLACKLEGVKNGEDPNLTLFHSGKSDDSSKIINTGICSRLIDGISIAVDDQRSETAGVLSPETPSIESHRNYPMKSRRKGIPQRAPF
AtPLATZ5	AT1G76590	MGPMMMRAEEDNYNLNPPPWLIPMLRANYFIPCSIHAASNKSECNMFCLDCSSEAFCSYCLLNHRNHRVLQIRRSSYHNVVRVNEIQKYIDISCVQTYIINSARIVFLNERPQPRIGKGV

		TNTCEICCRSLLDSFRFCSLGCKLGGMKRDPSLTFSLRGKHGREYEGEWESDEATTPTKI<br>RKTCAFNRLMSGLSISTVKCDYLSGDQPSSSSGDESGFKLSPGTPPIYNHRNSSRRKGVP<br>HRAPF
AtPLATZ6	AT2G01818	MNLSEKRRSEEVWIETLLNSEFFGICMNHKYLRKNEKNVFCIDCNVEICRHCCNTVTDSH<br>FLHRRLQICKYVYQDVIRLLEIQNYFDCSEIQTYKINGEKAIHLNSRPQAKDARPSTKAK<br>NGASCVTCKRYIQDHPNLFCSISCKISTPSKKHKFCFSPKLEQSVLEKEHSTQEGSLEEK<br>KSCTSSLTDVSEDSEVLLSDFSFRPLLRILKRKGISRRSPFY
AtPLATZ7	AT2G12646	MGIQKPAWLDALYAEKFFVGCPYHETAKKNERNVCCLDCCTSLCPHCVPSHRFHRLLQVR<br>RYVYHDVVRLEDLQKLIDCSNVQAYTINSAKVVFIKKRPQNRQFKGAGNYCTSCDRSLQE<br>PYIHCSLGCKVDFVMKRYRDITPFLKPCHTLTLGPDYIIPQDLLTDDEVAAYETPRSTVV<br>DGDESMSWSSASSDNNNAGAAAAYAATTTHVVRKKRTGFCLCAKSANSYKEVSEDPDDIS<br>ACINRRKGVPQRSPLC
AtPLATZ8	AT2G27930	MEEPKWLEGLLRTNFFSICPRHRETPRNECNMFCLSCQNAAFCFYCRSSFHIDHPVLQIR<br>RSSYHDVVRVSEIENALDIRGVQTYVINSARVLFLNERPQPKNSSHGAASSTPKTMSYFC<br>ETCCRTLLDPFRFCSLGCKVEGMRKNKEEEEERLRKERQQETHKGTHPPTHTSNSRRRKG<br>IPHRAPFAS
AtPLATZ9	AT3G50808	MSQYMDISGIHLYSINGFPIVYINQRRGNNNHRSRSNVMHKCKICEWEIDAASSALFCSM<br>ECKFRSVLGSQLDELMENSSEVTEISEEIDEPVMKKRHRRKGSPHRAPFF
AtPLATZ10	AT3G60670	MESGEFPAWLEVLLKDKFFNACLDHEDDKKNEKNILCIDCCLTICPHCLSSHTSHRLLQI<br>RRYVYRDVLRVEDGSKLMDCSLIQPYTTNSSKVVFINERPQSRQFRGSGNICITCDRSLQ<br>SPYLFCCLSCKISDVIMRQRGLSGFLRVCNVLDLTDEVTTTTPSSTLEPTGSNRTSSESS<br>GNEGEDMFWCQALACTATTEIVRKKRSSLSTTCRRVTEVVSTTNTEAPVNFLNRRKNPPQ<br>RAPLY
AtPLATZ11	AT4G17900	MAIEDQENTIREIKPKNRRIMGAGGPEEEENRWPPWLKPLLKEQFFVHCKFHGDSHKSEC<br>NMYCLDCTNGPLCSLCLAHHKDHRTIQIRRSSYHDVIRVNEIQKYLDIGGIQTYVINSAK<br>VVFLNERPQPRPGKGVTNTCKVCYRSLVDDSFRFCSLGCKIAGTSRGFEKGRENLLMETE<br>DSSSSIAIGKNITNLQSFSPSTPPLTTSSNCRIVKRRKGIPHRSPMG
AtPLATZ12	AT5G46710	MAIEDYENPNREIKPKNRRFMEGENQWPIWLKPLLNQHFFAQCKFHGHLPRTECKMYCLD<br>CTNDSFCSLCLSEHENHRTIQIRISSYHNVTKVDEIQKYLDISSIQTYVINSSKVLFLNE<br>RPQSKPGKGFTNACMVCYRGLAENCFRFCSIGCKVAGTSGVFQKRVKHTTNDSDNSNNSS<br>GVENNSSGAENGNSNLQSLSPPTPQFPPRSLRKRLRKGIPHRAPFS
BrPLATZ1	Bra003715	MMRAQEEEKEEEEEEEGHYRSPPWLIPMLRANYFVPCSIHAGSNKSECNMFCLDCTSDAF<br>CSYCLVNHKNHRVLQIRRSSYHNVVRVNEIQKYIDISCVQTYIINSARIVFLNERPQPRI<br>GKGVTNTCEICFRSLLDSYRFCSLGCKVNRIYSLCCIPVSVSLLN
BrPLATZ2	Bra007550	MGLALILQMETREFPAWLEVLLKEKFFNACLDHEEEKKNEKNILCIDCCLSICPHCLPSH<br>TSHRLLRIRRYMYNDVLRVEDGSNLMDCSLIQPYIVNSSKVVFINERPHSRQFRGSGNFC<br>NTCDRSLQSPYLFCSLSCKISDVIMRQRGLSGFLRVCISLNLTDDEGGVDMFLCQALACT<br>AATEIVRKKRSSLSSTCRRVTTAVSSANTEAPANFFNRRKNTPPQRAPLY
BrPLATZ3	Bra007562	MANVLVFLSTSTVRVQLLLLVLTSQRRKIIDGRLKPLLKEQFFVYCKFHVDSHKGDCNMY<br>YLDCTNGELCSLCLAHHKDLLTIQSKCVLAIILISFFGWLCYVVRFCLCIDWFWQIGRSL

		GLYRSISIACHSSWIHNLTIFYPSSNQRISTYKKGCLEKVIGGDSGGGSQHHYGTETHRK<br>KKRYHHHTAQQIQRLESSFKECLHPDDKQRNQLSRELGLAPRQIKFRFQNIRTQLNAQHE<br>RADNNALEAEKDKIRCENIAIREALKHAICPNCGGPPVNACLFQLN
BrPLATZ4	Bra011992	MEEPKWLEALLRTNYFNICPRHCETPRNECNMFCLSCQNAAFCIYCRTSLHIDHPVLQIR<br>RSSYHNVVRVSEIEKVLDIRGVQNYVINSARVLFLNERPQPKNSSHGPTSSTTKTISYFC<br>ETCCRTLLDPARFCSLGCKVEEMRKNKEDEEERLRKERQQETHKGTHPPTHTSNSRRRKG<br>TPHRAPFAS
BrPLATZ5	Bra012263	MIRIEEEEDYMSPPWLMPMLRGSYFVPCSIHADSNKNECNLFCLDCAGTAFCSYCLVKHK<br>DHRIVQIRRSSYHNVVRVNEIQKYIDITCVQTYIINSAKIVFLNERPQPRIGKGVTNTCE<br>ICCRSLLDSFRFCSLGCKLGGMKRGDQSLTFSVRGKHGREYQGGGSESDEATTPTKMRKT<br>NAFNRLMSGLSISTVRFDD
BrPLATZ6	Bra012632	MAIDDQENTIREFKPKNRRIMGAGGPEEEDNRWPPWLKPLLKEHFFAHCKFHVDSHKSEC<br>NMYCLDCTNGPLCSLCLAHHKDHRTIQIRRSSYHDVIRVNEIQKHLDISGIQTYVINSAK<br>VVFLNERPQPRPGKGVTNTCKVCYRSLVDDSFSFCSLGCKIAGSSRGFEKGRKNLLMETE<br>DSGCFSPSTPPLTASSSCRIAKRRKGIPHRSPMG
BrPLATZ7	Bra013149	MFILTPCKCVESLVRSIDTNMDIRSYRIVGIQKPAWLDALYAEKFFVGCPYHETAKKNEK<br>NVCCLDCCISLCPHCVPSHRYHRLLQVRRYVYHDVVRLEDLQRLIDCSNVQAYTINSAKV<br>VFIKKRPQNRQFKGAGNYCTSCDRSLQEPFIHCSLGCKVEFVMKSYGDITPFLKPCHSLT<br>LGPDYIIPQDLLADDDMAAYETPRSTVVDGDESMSWSSTSSELRDAATTTHVVRKKRTGF<br>CFCAKSANSYKAVSEDPDDISACINRRKGIPQRSPLC
BrPLATZ8	Bra013266	MAIEDQEKTIREIKPKNRRIMGAGGPEEEDNRWPPWLKPLLKEQFFVHCKFHVDSHKSEC<br>NMYCLDCTNDPLCSLCLSHHKDHRTIQIRRSSYHDVIRVNEIQKYLDISGIQTYVINSAK<br>VVFLNERPQPRPGKGVTNACKVCYRSLVDDSFRFCSLGCKIAGSSRGFEKGRRNLAMESE<br>DSGGGIGIGRNISNLQSFRPSTPPLTTSTSCRIAKRRKGIPHRSPMG
BrPLATZ9	Bra014478	MGLGSILQMEPKDFPAWLEVLLKEKFFNACLDHEDVKKNEKNILCIDCCLSICPHCLPSH<br>NSHRLLQIRRYVYNDVLRVEDGSKLMDCSLIQPYITNSSKVVFINERPHSRQFRGSGNFC<br>YTCDRSLQSPYVFCSLSCKISDVIMRQRGLSGFLHVCNFLHLTDEGATSTTPSSTLEATE<br>SDGDVGVDMFWCQALACTASTEVVRKKRSSLSATCRRVTTAVASTNTEAPVNFFNRRKNT<br>PPQRAPLY
BrPLATZ10	Bra014899	MVREGQEEEEEEEMIMTVAATTKPAWLEGLIAETFFSSCGIHESRRKSEQTVFCLLCCLS<br>VCPHCLPSHRSHPLLQVRRYVYHDVVRLSDLEKLIDCSYVQPYTINGAKVIFINQRPQSR<br>AKVSSNVCFTCDRILQEPFHFCSLSCKVDYLIYQGDDLSSILYRIDESDFTFASLRMDGH<br>DQFGEISTMVDDADDIMVISDQSEQGNNSNKKEKKKKKKKESNYFPGMVLSLGSRRKGAP<br>HRAPFS
BrPLATZ11	Bra015731	MMRAEEESPPPWLIPLLRANYFVPCSIHADSNKSECNMFCLDCTSSSFCSYCLTNHKNHR<br>VLQIRRSSYHNVVRVNEIQKYIDISCVQTYIINSARIVFLNERPQPRIGKGVTNTCEICC<br>RSLLDSFRFCSLGCKLGGMKRGNQSLTFSLKGKHGREYQAGEDSDEATTPTKIRKTCAFN<br>RLMSGLSISTAKSDYFSGDQWSSSSGDESGFNLSPGTPPIYNHRNSSRRKGVPHRAPF
BrPLATZ12	Bra016441	MIRTEEEEDYTSPPWLMPMLRGSYFIPCSIHADSNKNECNMFCLDCAGAAFCSYCLVKHK<br>DHRVVQIRRSSYHNVVRVNEIQKYIDISCVQTYIINSARIVFLNERPQPRIGKGVTNTCE<br>ICCRSLLDSFRFCSLGCKLGGMKRGDQSLTFSMKGKHGRDYQGGLESDEATTPTKLRKTK

		AFNRLMNGLSISTVRFDDYGPGGDQRSPNSGDEGGFSFSPGTPPIYNHRNSSRRKGIPHR<br>APF
BrPLATZ13	Bra017531	MAIEDHENPNREINPKNRTVMGGGGGEEEEGKWWPSWLKPLLKEPFFVQCNFHGHSPKSE<br>CNMYCLDCTNGSLCSLCLAHHKDHRTIQIRRSSYHDVIRVSEIQNHLDIFSIQTYVINSA<br>KVVFLNERPQPQPRPAKGVTNACNVCYRGLVDDCFRFCSLGCKVAGTSRSFRKRVKNAEM<br>ESENSSNSSGVEDNIPNPQSLTPSTPQLPSSTSLRKRPRKGIPYQSPLQ
BrPLATZ14	Bra021029	MAIEDQENTIREIKPKNRRIMGAGGPEEEDNRQPPWLKPLLQEKFFGHCKFHVDSHKSEC<br>NMYCLDCTNGPLCSLCLAHHKNHHTIQIRRSSYHDVIRVNEIQKHIDISGIQTYVINSAK<br>VIFLNERPQPRPGKGVTNTCKVCYRSLVDDSFRFCSLGCKIAGASRGFEKGRKNLVMESD<br>DSSSSIGIGKNIQSFSPSTPPLTASSHCRIAKRRKGIPHRSPMG
BrPLATZ15	Bra022055	MAIENHENPNRRRSMGGGGPEEEENQWPPWLKPLLKENFFAQCNFHGHSPKNECNMYCLD<br>CTNGGSLCPLCLEHHKDHRTIQIRRLSYHDVIRVNEIQMHLDILSVQTYVNNSAKVVFLN<br>ERPQLERVRGVRVTNACDVCSRGLADDCFCFCSLGCKVAGASRSFEKGVKHTLTELESSN<br>NSSGVEDSIPSLQSLITSTPQRPTSTSLRKRPRKGIPHRSLLQ
BrPLATZ16	Bra022058	MAIENHENPNQEIKPKSIRSMGVGGPEEEENQWPPWLKPLLKEEHFFVQCDSHGDSPKNK<br>CNMYCLDCTNGSFCPLCLEHHKDHRTIQIRRLSYHNVIRVNEIQMHLDILSVQTYVIDGA<br>KVVFLNERPQLERVRGVRVTNACDVCSRGLADDCFCFCSLGCKVAGTSRSFEKGGEHTVM<br>ESENSSNISGVENNIPNPQSVNASTTVLPTSTSLRKRPRKGIPYRSPLQ
BrPLATZ17	Bra023155	MVRGDHEEGEEEMMVAATKPAWLEGLMSETFFSSCGIHGSRRKSEKNVFCLLCCLSVCPH<br>CLPSHRSHPLLQVRRYVYHDVVRLSDLEKLIDCSYIQPTRRRSCLPHAVTTHCHLRCHRT<br>RAAATRHHLLSVCHQRTLENKPKLTTTETLTPPPEIPSRRKQRSSDPRNHPTKPPPNPST<br>TGAPPSRGADKLRRGFTTPLAPSLLRNHRRQGKSEPVRDTTRTRSEAKVDYLVYQGDDLS<br>SILYRIDESDFTFLSLRMDGHDQLGEISTMEDETDDIVVISDQSEQGNNSNKEKRKKKKE<br>SNYLPGMVLSVGSRRKGAPHRAPFS
BrPLATZ18	Bra023280	MKRTGPSSGIIVIRHQVISVDRHFLISLTASSRELYNSLFSKKLGIEENNQATTVRESKP<br>TSKRIIMGAEEEKNKMWPPWLKPLLREKFFVQCKLHADSHKSECNMYCLDCTSGPLCSLC<br>LSFHKDHHAIQIRRSSYHDVIRVSEIQKFLDITGVQTYVINSAKVVFLNERPQPRPGKGV<br>VNTCEVCYRSLVDSFRFCSLGCKISGISRSFDKKKKDWTNNLSDSNDSYSSSTSIGRLKK<br>NGDMIHNSFTPSTPPLSAVYRRIAKRRKGIPHRAPLGGLIIEY
BrPLATZ19	Bra025003	MAIEDYENPHRVIKPKNRRIMGADGLEEEENHRWPAWLKPLLKEHFFVQCNVHSPKSECK<br>MYCLDCANGSLCALCLAHHNNHRTIQIRRSSYHDVIRVNEIQKYLDIFGIQTYVINSAKV<br>VFLNERPQPRPGKGVTNTCKVCYRGLVDDCFSFCSLGCKVAGTARSFEKRVKPTPMEPDN<br>SSSNSSGVEDNIPNAQSLAPSTPHLPTSTSLRKRKRKGIPFQSPLQ
BrPLATZ20	Bra025865	MIRTEEQEDDYMSPPWLMPMLRGSYFVPCSIHADSNKNECNLFCLDCAGNAFCSYCLVKH<br>KDHRVVQIRRSSYHNVVRVNEIQKYIDISCVQTYIINSAKIVFLNERPQPRIGKGVTNTC<br>EICCRSLLDSFRFCSLGCKLGGMKRGDQSLTFSLRGKHGREYQGGSESDEATTPTKLRKT<br>NAFNRLMSGLSISTVRLDDYGPGGDQRSLSSGDEGGFSFSPGTPPIYNHRNSSRRKGIPH<br>RAPF
BrPLATZ21	Bra034379	MEEPKWLEGLLRTKFFNICPRHRETSRNECNMFCLSCQNAPFCIYCRSSLHIDHPILQIR<br>RSSYHDVVRVSEIEKVLDIREVQTYVINSARVLFINERPQPKNSSHGAASSTTTKTISYF<br>CETCCRTLLDPFRFCSLGCKVEGMRKNKEDEEERLRKERQQETHKGTHPPTHRTSNSRRR

		KGIPHRAPFAS
BrPLATZ22	Bra035494	MGAEEENSKSWPPWLKPLLQEKFFVQCKLHAYSHKSECNMYCLDCTNGPLCSFCLSFHKD<br>HHAIQIRRSSYHDVIRVSEIQKFLDITGVQTYVINSAKVVFLNERPQPRPGKGVVNTCQV<br>CYRSLVDSFRFCSLGCKISGTSKNFDKKRKDWTNNLSDSDDSYSSTTTSNGRLKKNTDMI<br>NNNSFTPSTPPLSAVNGRIVKRRKGIPHRAPFGGLIIEY
BrPLATZ23	Bra037203	MMGKGKNGIMKPAWLDALYAEKFFVGCPYHETAKKNEKNVCCLDCCISLCPHCVPSHRYH<br>RLLQVRRYVYHEVIRLEDLQKLIDCSNVQAYTINSAKVVFIKKRPQNRQFKGAGNYCTSC<br>DRSLQEPFIHCSLGCKVEFVMKRYRDITPFIKPCHTLTLGPDYIIPQDLHADDNMAAYET<br>PRSTVVDGDDSMGWSSTSSELGDAATTTHVVRKKRTGFCFCAKSANSYKIVSEDPDDISN<br>CINRRKGIPQRSPLC
BrPLATZ24	Bra038403	MMREGHEEEEMMMMLAKKPAWLEGLMAETFFSSCGIHESRRKSEKNVFCLLCCLSVCPHC<br>LTSHRSHPLLQVRRYVYHDVVRLSDLEKLIDCSYIQPYTINGAKVIFINQRPQSRAKVSS<br>NVCFTCDRILQEPFHFCSLSCKVDYLQYQGDDLSSILYRIDESDFTFSSLRMDGHDQLGE<br>ISTMEEDTDDIMVMSDQWEQGNNSNKKEKTKKKKESKYSPGMVLSLGSRRKGAPHRAPFS
OsPLATZ1	LOC_Os01g33350	MKGEFVPPWLELLLSTQFFNTCTSHHNSPRNECNLFCIDCQAPEAAFCYYCRSCHHSSHR<br>VIQIRRSSYHDVVKVSELEDILDISDVQTYVINSARVVFLNERPQLRGCGVLAIKSSPSS<br>LSSYNCETCSRVLLDAFRFCSLGCNVSYKPLQQVLFKLILLCNLICSQILG
OsPLATZ2	LOC_Os01g33370	MKGGESVPSWVELLLSTQFFTTCSSHLISPRNECNFFCIDCQTPQASFCYYCRLSHHSSH<br>HVIQIRRSSYHDVVKVSELEDILDISDVQTYVINSSRVVYLTERPQLRSCGVSNTKLSSS<br>QTYKCEICSRTLLDDFRFCSLGCNFAAIKRDNEKNVAQNGIASNANEVKIGTNNGSTNAG<br>SANEISSDANNYRNEIPSSTRVIRHRRKGIPRRAPFF
OsPLATZ3	LOC_Os02g07650	MGMRPGWVGGLVEESFFVGCPAHESRKKNEKNIFCLACCTSICPHCAPSHRHHPLLQVRR<br>YVYNDVVRLGDLEKLIECSYVQPYTINSAKVIFLKPRPQSRPFKGSGNVCLTCDRILQEP<br>FHFCSLSCKVDHVMVHGGGDLSNIILLHPHHHPNTATASAFPRFEDLRVGADDDAAAITA<br>VTPEGRYGGGGGGSSDNGGGDGGGGEVGEVKRKKKKGGGFFPQILGLGSRRKGAPHRSPL<br>S
OsPLATZ4	LOC_Os02g09070	MWKPAWLEALNTQKFFIACSFHEHAKKNEKNICCLDCCTSICPHCVAAHRVHRLLQVRRY<br>VYHDVVRLEDLEKLIDCSSVQSYTINSSKVVFLKKRPQNRQFKGSGNICTSCDRSLQEPY<br>FHCSLDCKVEYILRQKKDLSAYLRPCKTLQLGPDFFIPHDADDETTHSTLVDVDEPMGSS<br>DSENLSAPCPNFVRKKRSGPYICARSANRVSDDDMATNMSRRKGVPHRSPLC
OsPLATZ5	LOC_Os02g10000	MAAGLAEREPAWLRSLLGARFFEACAAHRGMSRNECNQYCLTCAAAADDAGGAAAVGCQW<br>CVVAAHGGGAGRDRGHRHRVVQVRRSSYHNVVRVSELERTLDLTRVQTYVINRDRVVFLN<br>ERPQAPRNGRCAAAAAVACAACEACGRGLLDVAFRFCSLGCKLKCMESDPTLTFTIDPNN<br>IPEPQISGPQEDEEEDDDEEDEPFYPTKANAAQSKAAGGGGCRPPPPPSASSSRPRRGGR<br>RVARGDKEEDQEAAANILAFAAAAARSVPAASAADPNSYRRRARKGAHRAPERSPFF
OsPLATZ6	LOC_Os02g44260	MQSPAVRGAPQWLRGLLSEEFFDSCGAHPGERKNDKNHFCVDCAAALCRHCLPHDASHGV<br>LQIWKYASCFVVRVDDLKLFDCNGIQTYCTDHESYSYMVSGVQSHTLSDHEVVFLNERTA<br>RKRSASVENPCAACARPLPSGHDYCSLFCKVKHLGESDQGLRRALRVNRRSAAAAGGGGG<br>GEDPAVAEASQSGKRRASSSESGRSCGGTLRKRSRKQPAPARSPSC
OsPLATZ7	LOC_Os02g46610	MAIDHASPFSLKNRGDMGGRGYEEEEVENQRWPPWLKPLLSTSFFVQCRIHADAHKSECN<br>MYCLDCMNGALCSLCLSHHRDHHAIQIRRSSYHDVIRVSEIQKVLDITGVQTYIINSARV

		VFLNERPQPRPGKGVTNTCEVCERSLLDSFRFCSLGCKIVGTSGGYRPRKKHGGCGGGGG<br>GGDGGKKKKKRAALKDARYESEDSCTSTSGGSSDKSSVVQSFTPLTPPPTSASYRTGNKR<br>RKGVPHRSPFGSLIVEF
OsPLATZ8	LOC_Os03g12440	MLSKMHMKWFSFLEWPSATFASSFIKTQSSLAQYYLLMFVKIIELCKLKGKETRAEPEVS<br>SVSKASGGSSEHINKLPPPVPVQEEEEAPEWLDVLLRTKFWGQCKQHWDASRAEVCIFCL<br>RCRQVLCPRCSHDEPGHRLLKVRRYMYRSVVLARDLQGLNVDVSRVQTYIVNGQKGVHLR<br>PMRRSPQFKPHVGVDISQDDFSGPEAERRHKQTLGIVVESSPQQSIPQPFDASPVRNEDA<br>TMVEAECGQVQTNATESESSAVGDADEVIPKVTKFNVDIHSLRRRVRKQAAPQRAPFF
OsPLATZ9	LOC_Os04g50120	MAIDDESPLRINTTRGGAMGGGGECDGAENQRWPPWLKPLLATSFFGQCKLHADSHKSEC<br>NMYCLDCMNGALCSQCLSYHRDHHAIQIRRSSYHDVIRVSEIQKVLDITGVQTYIINSAR<br>VVFLNERPQPRPGKGVTNTCEVCERSLLDTFRFCSLGCKIVGTSGDYRGRKRHAGGGIKK<br>TKKLHKGAAAVPSDSDDSSTTTSGGSDKSSVVQSFTPSTPPATANSYRTGKRRKGVPHRS<br>PFGSLMVEF
OsPLATZ10	LOC_Os06g41930	MVMVGMMETSPDDGGGGEAMAAAQQTEEDMGPPWLRPLLSTSFFVACASHPELSKNECNL<br>FCLGCTGDALCAYCLPAHRDHHVVQIRRSSYHNVIRVSEVGKLIDISHVQTYVINSAKIV<br>FLNGRPQARPGKGVTNTCEICCRSLPDSFRFCSLGCKLGGMRWDPSLTFAIRPKRGQDSG<br>DGGSGSDYDSFSPKKARRAAAGYDQLGRFDRGMIRWSDDEGSKSNTAPITPTTPPISRCR<br>PSRRKGIPHRAPFYG
OsPLATZ11	LOC_Os06g45540	MGMRPGWVGGLVEESFFVGCPAHESRKKNEKNIFCLGCCASICPHCAPSHRHHPLLQVRR<br>YVYNDVVRLDDLDKLIDCSFVQPYTINSAKVIFLKPRPQSRPFKGSGNICLTCDRILQEP<br>FHFCCLSCKVDHVMMQGGDLSNILYMSGGSSGEPDLAAGFPRFENLRVDGGGGGGGGLSD<br>DDDDHQVTTPNSILEDPLHHHHHQYYGGGGSSNNGRSTSPAPTTADVPRKKKSGGGGGFF<br>PQIVLSLNNRRKGAPHRSPLA
OsPLATZ12	LOC_Os08g44620	MSEGAAGTTRLPEWLETLLSTRFFLACGAHPASPRNECNMFCLDCPSPSPPFCYYCRSHR<br>HQSHRVIQIRRSSYHDVVRVTEVEDVLDISGVQTYVINSAKVLFLNERPQPRGAGAAAGK<br>AAASPYNCQICARALLDPFRFCSLGCKLVDTKTGGRGATVQPGDATNDDAAAAGGSSKNG<br>GARPQGRRRKGIPQRAPFGS
OsPLATZ13	LOC_Os09g02790	MLPSPDDSSSSSPSSKTNPSEKGEEITVVEEPILFDNNQEEDNSSNPLNLLADTSELFRN<br>MDDDESPLWLNVLLRTIFWRKCDVHEQLENAHRAEESIFCINCLKTICPHCTHDEPSHQL<br>LKVRRYIFRSVVRVKDMQNFGIDMSYIQTFKCNGHKVVHLRPIKRSEHHRPKAGTPHCTS<br>CHCWLHNAPSLTCSLSCKKKAGISSDDFSGPEASTRVSRSRNHASNVNQKHPTNTKLRKK<br>PRKQANPERAPFF
OsPLATZ14	LOC_Os10g42410	MAIDHESPFKELRLKNRRIMGGGGPEPEEEEAVAHGEQWPRWLSPLLSASFFSQCKVHAD<br>SHRSGECNMFCLDCAADADAAAAALCSLCLAHNHRDHHTIQIRRSSYHDVIRVSDIQRFM<br>DIGGVQTYVINSARVVFLNERPQHKAGKGAVANICEVCSRSLLDNFRFCSLGCKVVGCSP<br>HAATAAATATATAARRKRLRHAHAMASTSDSDNSTSPAKRSFTPSTPPPPPTLPPKRRKG<br>IPHRAPFGSLIVEY
OsPLATZ15	LOC_Os11g24130	MGRRVHLLRWGHDSGPGILMIRRSSYNDVVRVTEVEDVLDISGVQTYVINSAKVLFLNEH<br>PQPRDASTVVGEAAASPYNC
ZmPLATZ1	GRMZM2G004548	MAIDHAARLGLTSRGATGGGGCGDDVDAENRRWPPWLKPLLCTSFFVQCRIHADAHKSEC

		NMYCLDCMDGALCSLCLARHRDHHSIQIRRSSYHDVIRVSEIHKVLDIAGVQTYIINSAR<br>VVFLNERPQPRPGKGVTNTCEVCERSLLDCFRFCSLGCKIVGTARGYRPKKKHGGGGGG<br>NKRKRAALKDVRSDSEESCTSTSGASSDKSSVVQSFSPSTPPPASASYRRPGNKRRKGVP<br>HRSPFGSLIVEF
ZmPLATZ2	GRMZM2G006585	MKRETMPSWLELLLATQFFTTCANHLLASRNECNLFCTQCETKPAAFCNYCRSSDHSTHR<br>VIQIRRSSYHDVVRVSEIEDILDVSDVQTYVINSARIVFLNERPQLRASSVPICKAPTSS<br>THSCETCSRVLLDAFRFCSLGCNLRGLNMEAGMQAMVGNNPRSNGMDHVARIDNVGSSTT<br>NDQNSCNDKNYEEPPPKRVARHRRKGIPQRAPFF
ZmPLATZ3	GRMZM2G017882	MFSANCYVRDQVRRYVYHDVVRLGDLEKLIDCSCVQTYTINSAKVIFLKPRPQSRPFKGS<br>GNICLTCDRILQEPFHFCSLSCKVDHVMTQGGDLSNILQHYGAGGGGGGGTADPDRLAFP<br>RFENLRVVDGSDLDDDVQVVTPDSTLEDPTNNAGGGSSDNGTDDARRQVVVHGGGEAAKR<br>KKGGGFLPQIVLSLGGGGGGGNRRKGAPHRSPLA
ZmPLATZ4	GRMZM2G070295	MIMAMWKPGWLEALDTQKFFVACSFHEHAKKNEKNICCLDCCTSICPHCVAAHRAHRLLQ<br>VRRYVYHDVVRLEDLEKLIDCSSVQSYTINSSKVVFLKKRPQNRQFKGSGNICTSCDRSL<br>QEPYFHCSLDCKVEYILRQKKKLSAYLRPCKTLQLGPDFFIPHDADDDTTHSTLVDVDEP<br>MGSSDSENLSVPCTNFVRKKRSGPYICARSANRVSEEDMATNMSRRKGVPQRSPLC
ZmPLATZ5	GRMZM2G077495	MSCIGGSSSSTASNKRKGKEIVVEVSAVVEEEEKQQQQRKGKEVALEEVPLPAVAESYD<br>DSDLDSGSGWDFYEEEYSEKKEQERKEKKPAWLDTLLRTKFWDPCKEHGSKNRADQCMFC<br>LRCSKLSCPRCVHDQPGHRLLKIRRYVYRSVVHASDMQELGIDVSRIQTYVINARKVLHL<br>RPMNRSKHFRPQAGTPRCITCRTWLRSAPNLFCSLTCEEDVDVSQDDFSGPEAELRYRSF<br>QVHMAEPAEELLPDDPEVEHEIMPAQVEPPPLAAAAAANQNVSLRRRARKQAAPLRAPFF
ZmPLATZ6	GRMZM2G086403	MAIDDESPIRVNSRGGGAMGGGECDGAENQRWPPWLKPLLGTSFFGQCKLHADAHKSECN<br>MYCLACMNGALCSQCLAYHRDHHAIQIRRSSYHDVIRVSEIQKVLDISGVQTYIINSARV<br>VFLNERPQQRPGKGVTNTCEVCQRSLLDTFRFCSLGCKIVGTSGDLRIRKKQAVVKKHQK<br>KKKQQAQQHRGAALDSEDDSSSTSTSRGSDRSSVVQSFTPSTPPATANSFRTGKRRKGVP<br>HRSPFGSLMVEEF
ZmPLATZ7	GRMZM2G091044	MAIDHATPLGLKRRGAMGGGGECDDGSDTRRWPPWLRPLLSASFFVQCRVHADAHKSECN<br>MYCLDCMGGALCALCLAARHRDHHSIQIRRSSYHDVIRVSEIQKVLDIAGVQTYIINSAR<br>VVFLNERPQPRPGKGVTNTCEVCERSLLDCFRFCSLGCKIVGTARGYRPGKKKHGGGGGN<br>KKRASPAPALKDVRSDSEDSCTSTSGASSDKSSVVQSSPPPPTSASHRPPGNKRRKGIPH<br>RSPLGSLIVEL
ZmPLATZ8	GRMZM2G093270	MAIDHESPFKELRLKNRRIMGGGGPDPEPEEEEEEGVTAAYAERWPRWLQPLLSARFFAQ<br>CRTHSDSNRSGECNMFCLDCSAAGGTGTGAGALCSLCLAQHGHRDHHTIQIRRSSYHDVI<br>RVSDIQRFMDIAGVQTYVINSARVVFLNERPQQQKPGGGKAASSSSASANLCEVCARSLL<br>DNFRFCSLGCKVVGCSPDAAKARNWLLRAADGDGSTTSSSAPRNADRKLSFTPPTPQPTL<br>PTKRRKGIPHRAPFGSLIVEY
ZmPLATZ9	GRMZM2G094168	MFCLDCSAAGAGAGARALCSLCLAQAHRDHHTIQIRRSSYHDVIRVSDIQRFMDIAGVQT<br>YVINSARVVFLNERPQQQRPGCKAASASANLCEVCARSLLDNFRFCSLGCKVVGCSPDAA<br>KARSWLLRPAVGGSGDGDSTSSSPLRDAQKRQSFTPPTPQPAKRRKGIPHRAPFGSFIVE<br>Y
ZmPLATZ10	GRMZM2G131280	MAIDDESPLRVNTRGGAMGGGERDGGENQRWPPWLKPLLGTSFFGQCKLHADAHKSECNM

		YCLDCMNGALCSQCLAYHRDHHAIQIRRSSYHDVIRVSEIQKVLDISGVQTYIINSARVV<br>FLNERPQPRPGKGVTNTCEVCERSLLDTFRFCSLGCKIVRTSGDFRIRKKHAIVAKKKRE<br>KKHAPQQKQHRGAADSADDDDDDSSTSTSGGSDKSSVVQSFTPSTPPATTANSFRAGKRR<br>KGVPHRSPFGSLVVEF
ZmPLATZ11	GRMZM2G171934	MMPRRVASVPVPDWLEALLATRFFLACAAHPASPRNECNMFCLDCTGAPPPPPPAFCYYC<br>RAHRHSSHRVIQIRRSSYHDVVRVSEVEDVLDISGVQTYVINSARVLFLNERPQPRGAGA<br>AAGKAAASPYNCEICGRALLDPFRFCSLGCKLVDTKRSNGHAASSADGGGGGGGAASGND<br>ETTEAGGSKNGPGARPHGRRRKGTPHRAPFWS
ZmPLATZ12	GRMZM2G311656	MGKETVVEVHGAVAEEKQQVRNGEETAAEDDSGGSDPDSDSESDLDSESDWDSYSEEEEE<br>RQQQRKKKEKPKNKKKKKKQRRRQQQQQPAWLITLLRTRFWEPCKEHVSKNRAEQCMFCL<br>KCCKVTCPRCTHDLPGHRLLKIRRYVYRSVVHASDMQALGVDVSRIQAYVVNAKKVLHLR<br>PMSRSKHFRPQAGTPRCVTCRTWLRSAPNLFCSLACQGNVDVAQDDFSGPEAEVRYRSLQ<br>VQMAEPSGAAADELPGPEAAHEVPAQVPPPPPPAANQNASLRRRPRKQAAPERAPFF
ZmPLATZ13	GRMZM2G323553	MVSLREMARSDAERAPPAWLRALLETRFFDACPEHQANDAGRANRKRTSGCNFLCTHCAD<br>RALCSGCLGNHEGHGLIQIRRSSGNNVVKVDDVQNRLSVSLVQTYVVYNGDYAVFLNRRPM<br>SGHGKHGASHCEQCGRGLQDEDCRFCSLECKAKGIEDRLDFSVSFAVDPNNFSSSGDDTE<br>SDDDEDSSYPSKFQKLETIPASSSKPVASGGQHSIGKKQY
ZmPLATZ14	GRMZM2G342691	MHPVAARGAPHWLRGLLSEEFFDACAAHPGERKNDKNHFCVDCAAPLCRHCLPHEHVHDV<br>LQIWKYASCFVVRIDDLKLFDCTGIQSHTVSDHEVVFLNERTARKRSTSAENPCAACARP<br>LLPGHDYCSLFCKPRRRSPKPYLPSRNSSRRVRTKFELKLHRLILNGGLAGPGASAEDVA<br>DALRVPYLEFRRQKRGPFVASVRRALTSIPIPSPSSSDSSDDGSSGSRRRGHHDAHATTA<br>SSSTSVSDAVAHPSPPAPAYDVTKSMLRSQYAAQTPKRGQQLEIEVAAEKLRRLITADGG<br>GGGDAKPEAAPSSEGFGRGEVISRVSGGVVDMHLRKVRHLDTSKKSEFHKLLQLKDELSI<br>HVNSSKRYGTQLK
ZmPLATZ15	GRMZM2G408887	MTKRCTNLACGMLPSMVGIGGRVGRCRLQLLGAFHSRPSPPSYPLRPSLPQSSAVIAVSN<br>SCNIVDLHQRGVYSDVETLGTAVEPARRFTIYLVGPHRMRQGGGERDDAENQRWPPWLKP<br>LLGTSFFSQCKLHEDAHKSECNMYCLDCMNGSLCSQCLAYHRDHHAIQIRRSSYHDVIRV<br>SEIQKVLDISGVQTTCIGLLPGSFYCRLSIASVVSTLTASYACTFSNTMGF
SbPLATZ1	Sobic.001G288100	MAIDHDSPFKELRLKNRRIMGGGGPDPEPEEEEEEAATAAYAEQWPRWLQPLLSARFFAH<br>CKTHSDSHRSGECNMFCLDCSSAAAGAGTRALCSLCLAHGHRDHHTIQIRRSSYHDVIRV<br>SDIQRFMDIAGVQTYVINSARVVFLNERPQQQKPGCGGGGKAASASANLCEVCARSLLDN<br>FRFCSLGCKVIGCSPDATKARNWLLKAGADGDDSTSSSSALRNADKKQSFTPPTPQPTLP<br>TKRRKGIPHRAPFGSLIVEY
SbPLATZ2	Sobic.001G345600	MSSIGGSSNTASNKGKGKEIVVEVPSVADEKQQLSKGKEIVLESDGYESKWESDFTDSDP<br>KSESEEQEEQEKKPTWLEMLLKTTFWDPCKEHGSKNRADQCMFCLKCSKVTCPRCTHNKP<br>GHRRLKIRRYVYRSVVHASDMQERGIDVSRIQTYVINARKVLHLRPMNRSKHFRPQPGTP<br>HCITCGVWLRSAPNLYCSLVCEGNFDISKDDFSGPEAELRYRSLQVHMVQPPSEAAAEEL<br>TNSEVESVIHAQVVEPLPPMVANQNASLRRRARKQARPERAPFF
SbPLATZ3	Sobic.001G345700	MSSIGGSSSSASNKRKEKEIAVEEEKQQPRKGKEIVPVEESDGYEPDSDFELNSDSDLES<br>DSEEDEKEEEQQQQQQEKNKKPAWLEMLLRTKFWDPCKEHGSKNRAEQCMFCIKCFNVT<br>CPRCTHSMPGHHLLKIRRYVYRSVVHSSDMQDLGIDVSRIQTYVINARKVLHLRPMNRSK

		HFRPQAGTPRCITCRTWLRSRPNLYCSLVCEGNFDISQDEFSGPEAELRYRSLQVHMAEP<br>PSEAAVEELSDFEAEAAIPAQVVEPLPPPPAANQNTSLRRRARKQVKPERAPFF
SbPLATZ4	Sobic.001G345800	MSSIGGSSSAASNKGKEIVVEEERQQPMKGKEIVPEEESDGYESDFNLESDFDLVLDSEE<br>EQEQEQEQEQEQEQEQEKTKKPTWLEALLRTKFWDPCKEHGSKNRADQCMFCLKCFKVTC<br>PRCTHSKLGHRRLKIRRYVYRSVVHASDMQERGIDVSKIQTYVINARKVVHLRPMNRSKH<br>YRPQAGTPRCITCRTWLRSTPNLYCSLVCEGNFNISQDDFSGPEAELRYRSVQVHMSDEP<br>SGAAAEELPDSEAEPEMPAQVDEPPPLPVANQNASLRKRARKQAKPERAPFF
SbPLATZ5	Sobic.004G057700	MGMAPAGWVAGLVAESFFVACPAHESRKKNERNIFCLACCASICPHCAPAHRHHPLLQVR<br>RYVYHDVVRLGDLEKLIDCSCVQSYTINSAKVIFLKPRPQSRPFKGSGNICLTCDRILQE<br>PFHFCSLSCKVDHVMMQGGDLSNILQYYGGSGVAGDPDRLAFPRFENLRVDGSDLDDDTD<br>GGQVTPNSILEDPTQHYGNGGGGGSSDNGGDTRVDGGARRGGEAAKRKKGGGFFPQIVLS<br>LGGGGNRRKGAPHRSPLA
SbPLATZ6	Sobic.004G067800	MIMAMWKPAWLEALNTQKFFVACSLHEHAKKNEKNICCLDCCTSICPHCVGAHRVHRLLQ<br>VRRYVYHDVVRLEDLEKLVDCSSVQSYTINSSKVVFLKKRPQNRQFKGSGNICTSCDRSL<br>QEPYFHCSLDCKVEYILRQKKNLSAYLRPCKTLQLGPDFFIPHDADDDTTHSTLVDVDEP<br>MGSSDSENLSAPCTNFVRKKRSGPYICARSANRVSDEDMATNMSRRKGVPHRSPLC
SbPLATZ7	Sobic.004G076400	MVSLKEMARLDKERAPPAWLHTLLATTFFDACPEHQESEGCANRRTASCNFFCTHCAGHA<br>LCSSCLDNHEGHELIQIRKLSGHNAVKVDDVQHLLSVSFVQTYLYNGGYVVFLNRRPMYG<br>LGNRGVFHCEECERGLLDKAYHFCSFGCKAEGIEDRLDFNVSFAVNPNKDETELDDNEGS<br>FSEAGYHMSIV
SbPLATZ8	Sobic.004G076600	MVSLKEMARSDKERAPPAWLHTLLATTFFDACPEHLESEGCANRRTATSCNFFCTHCAGH<br>ALCSSCLDNHEGHELIQIRKLSGHNAVKVDDIQHLLSVSFVQTYLYNGGYAVFLNRRPMY<br>GLGNHGVSHCEECERGLLDKAYRFCSFGCKAEGIEDRLDFNVSFAVNPNKYETESDDNEG<br>SFSEAGYHMSIV
SbPLATZ9	Sobic.004G076666	MVSLKEMAWSDKERAPPAWLHTLLATTFFDACPEHLESEGCANRRTATSCNFFCTHCAGH<br>ALCSSCLDTHEGHELIQIRKLSGHNAVKVDDVQHLLSVSFVQTYLYNGGYVVFLNRRPMY<br>GLGNHGVSHCEE
SbPLATZ10	Sobic.004G275200	MRSRHGLVGLNQIRRSSYHDVIRVSEIQKVLDIAGVQTYIINSARVVFLNERPQPRPGKG<br>VTNTCEVCERSLLDCFRFCSLGCKIVGTARGYRPKKKHGIGGGGHKKKRAALKVKDVRSD<br>SEDSCTSTSGASSDKSSVVQSFSPSTPPPTSASHRRPGNKRRKGVPHRSPFGSLIVEF
SbPLATZ11	Sobic.004G293300	MQAAAMRGAPQWLRGLLSEEFFDACAAHPGERKNDKNHFCIDCAAALCRHCLPHEHAHDV<br>LQIWKYASCFVVRVDDLKVFDCTGIQSHTVSDHEVVFLNERTARKRSASAENPCAACARP<br>LLSGHDYCSLFCKVKHLGESEHGLRRALRVSRQEVAPTSEPQSGKRRPLSSSSSDSGPSC<br>SGSFRKRSRKQSEPTQAPFY
SbPLATZ12	Sobic.006G197800	MAIDDESPLRVNTRGGAMGGGGCDGAENQRWPPWLKPLLGTSFFGQCKVHADAHKSECNM<br>YCLDCMNGALCSQCLAYHRDHHAIQIRRSSYHDVIRVSEIQKVLDITGVQTYIINSARVV<br>FLNERPQPRPGKGVTNTCEVCERSLLDTFRFCSLGCKIVGTSGDFRIRKKHVVVTKKKKQ<br>AQHQHQHRGAAAASESEDDSSTSTSGGSDKSSVVQSFTPSTPPATANSFRTGKRRKGVPH<br>RSPFGNLMVEF
SbPLATZ13	Sobic.007G167300	MMLQRRVASAPVPDWLEALLATRFFLACAAHPASPRNECNMFCLDCRGAPPPAFCYYCRA<br>HRHSSHRVIQIRRSSYHDVVRVSEVEDVLDISGVQTYVINSARVLFLNERPQPRGAGAAA

		GKAAASPYNCEICGRALLDPFRFCSLGCKLVDTKRSNGHSAAAAADTDGGGAANGNDEAAEAAGGSKNGGPAARPQGRRRKGTPHRAPFWS
SbPLATZ14	Sobic.008G064400	MKREMVPSWLEILLATQFFTTCANHLLACRNECNLFCTQCEATPAAFCNYCRSINHSTHRVIQIRRSSYHDVVRVSEIEDILDISDVQTYVINSARVVFLNERPQLRASGVPICKAPSSSTHSCETCNRALLDAFRFCSLGCNLKGLNMEMSTPSMVENSPQSNGKDHVTMIDDVGSSTTSDKDSCNDKNNEEPPPKRVARHRRKGIPQRAPFF
SbPLATZ15	Sobic.008G066600	MKRETVPSWLELLLAAQFFTTCANHLLVSRNECNLFCTQCEATPTALCNHCRSSDHSTHHVIQIRRSSYHDVVRVSEIEDILDISDVQTYVINSARVVFLNERPQLRASGVPICKAPSSSTHSCETCNRALLGAFRFCSLGCNLRGINMERSTPAMVENNPQSDIKNHLTIDSVGSSTTSDKDSCNDNNNEEPPPKRVARHRRKGIPQRAPFF
SbPLATZ16	Sobic.010G196500	MVMVSVASPSPSPSPMVRSEEDLGPPWLRPLLGTSFFVPCRLHPELSKNECNLFCLGCTGDALCAYCLPAHRDHHVVQIRRSSYHNVIRVSEVGKLIDISHVQTYVINSAKIVFLNGRPQARPGKGVTNTCEICCRSLPDSFRFCSLGCKLGGMQWDPSLTFAIRPKRGQGSGDDGSGSDDDSFSPKKPRRMAGGFDLSRFERPGIRWSDDEGSRSNITPPMNRCRPSRRKGIPHRAPFYG
SbPLATZ17	Sobic.010G220800	MGMRPGWVGGLVEESFFVGCAAHENRKKNEKNIFCLGCCASICPHCAPAHRHHLLIQVRRYVYNDVVRLDDLERLIDCSFVQPYTINSAKVVFLKPRPQSRPFKGSGNVCLTCDRILQEPFHFCCLSCKVDHVMMQGGGDLSNILYVPGGGGGGPPDLVGCGFPRFENLRFDDDPAAQYGGQVTPNSILEDPMQHGGSSSGGSARNARRGDDVPTRKKSGGGGGFFPQIVLSLGNRRKGAPHRAPLA

Table S11. The primer sequences used in qRT-PCR analysis of the *SlPLATZ* genes.

Gene Name	Forward Primer	Reverse Primer
SlPLATZ1	CAAAGGCCACAAACAAGGGC	CTTCGTAAGTTTGCCCTCTGT
SlPLATZ5	AGTGGTTGTTTGGGGATGA TTTT	ACCTCGTGAAAGTAAAGGGAC
SlPLATZ10	TCCTACTGCTAGTTTTATATGTCTT	GTTGGCATACTCCTTCCAGG
SlPLATZ11	TTTGCCCTCACTGCCTTCCA	GAGCTTCTCTAGGTCATCCAA
SlPLATZ12	GGAGTGTAACTTGTGAAGCTTG	AGTTCTTCTGACGTCCTTTGC
SlPLATZ14	GGTAAGTCCTTCTTCTGTTTCA	CGATCAACACAACAATTTCTATGT
SlPLATZ17	AAATACTAGCGCCTTTCCTC	ACTTAAGTATTATCTAATCCCAAC
SlPLATZ19	GGATACATTTCGTTTCTGTTCTC	TTCCTCCTCTTGATGATATGCC
SlPLATZ20	GTAAGCTTTAGTTCCATTTCTC	CAAAAACATAATCAATTAGTAGATG

Table S12. Information of samples used to analyze RNA-seq data.

Run	Project	Sample_ID	Tissue	Development_stage
SRR12026415	PRJNA639840	S-H-C-1	leaf	Seedling stage
SRR12026416	PRJNA639840	S-H-C-2	leaf	Seedling stage
SRR12026417	PRJNA639840	S-H-C-3	leaf	Seedling stage
SRR12026418	PRJNA639840	S-1	leaf	Seedling stage
SRR12026419	PRJNA639840	S-2	leaf	Seedling stage
SRR12026420	PRJNA639840	S-3	leaf	Seedling stage
SRR12026424	PRJNA639840	H-1	leaf	Seedling stage
SRR12026425	PRJNA639840	H-2	leaf	Seedling stage
SRR12026426	PRJNA639840	H-3	leaf	Seedling stage
SRR7652567	PRJNA484882	DL-C-1	leaf	Seedling stage
SRR7652566	PRJNA484882	DL-C-2	leaf	Seedling stage
SRR7652565	PRJNA484882	DL-C-3	leaf	Seedling stage
SRR7652569	PRJNA484882	D-1	leaf	Seedling stage
SRR7652568	PRJNA484882	D-2	leaf	Seedling stage
SRR7652563	PRJNA484882	D-3	leaf	Seedling stage

SRR7652564	PRJNA484882	L-1	leaf	Seedling stage
SRR7652571	PRJNA484882	L-2	leaf	Seedling stage
SRR7652570	PRJNA484882	L-3	leaf	Seedling stage
SRR15607673	PRJNA756379	E117-1	inflorescences	Flowering stage
SRR15607672	PRJNA756379	E117-2	inflorescences	Flowering stage
SRR15607671	PRJNA756379	E117-3	inflorescences	Flowering stage
SRR15607670	PRJNA756379	M116-1	inflorescences	Flowering stage
SRR15607669	PRJNA756379	M116-2	inflorescences	Flowering stage
SRR15607668	PRJNA756379	M116-3	inflorescences	Flowering stage
SRR15607677	PRJNA756379	L117-1	inflorescences	Flowering stage
SRR15607676	PRJNA756379	L117-2	inflorescences	Flowering stage
SRR15607675	PRJNA756379	L117-3	inflorescences	Flowering stage
SRR12443441	PRJNA655574	GR-1	fruit	green ripe stage
SRR12443430	PRJNA655574	GR-2	fruit	green ripe stage
SRR12443419	PRJNA655574	GR-3	fruit	green ripe stage
SRR12443437	PRJNA655574	HaR-1	fruit	half ripe stage
SRR12443436	PRJNA655574	HaR-2	fruit	half ripe stage
SRR12443435	PRJNA655574	HaR-3	fruit	half ripe stage
SRR12443371	PRJNA655574	FR-1	fruit	full ripe stage
SRR12443370	PRJNA655574	FR-2	fruit	full ripe stage
SRR12443369	PRJNA655574	FR-3	fruit	full ripe stage

Table S13. The primer sequences used in subcellular localization analysis.

Gene Name	Primer sequence (5' to 3')
SlPLATZ11	GCAGCCCGGGGGATCCATGGAAGAGATGATGAAACCTGC
	CCATTCTAGAACTAGTAGAAAGTGGAGATCTTTGAGGGGC
SlPLATZ12	GCAGCCCGGGGGATCCATGCTAATCGGAGGTGAAATTCG
	CCATTCTAGAACTAGTACGTATCGGAGCTCTTCTAGG
SlPLATZ19	GCAGCCCGGGGGATCCATGACAATGCTGGTTCCGCC
	CCATTCTAGAACTAGTTGAACCAAGAGGTGCTCTATGAGG
